# Increased moist heat stress risk across China under warming climate

**DOI:** 10.1038/s41598-022-27162-2

**Published:** 2022-12-29

**Authors:** Shuai Sun, Qiang Zhang, Vijay P. Singh, Chunxiang Shi, Gang Wang, Wenhuan Wu, Zexi Shen

**Affiliations:** 1 State Key Laboratory of Earth Surface Processes and Resource Ecology, Beijing Normal University, Beijing, China; 2 Faculty of Geographical Science, Beijing Normal University, Beijing, China; 3 National Meteorological Information Center, China Meteorological Administration, Beijing, China; 4 Advanced Interdisciplinary Institute of Environment and Ecology, Beijing Normal University, Zhuhai, 519087 China; 5 Department of Biological and Agricultural Engineering, Zachry Department of Civil and Environmental Engineering, Texas A&M University, College Station, TX USA; 6 National Water and Energy Center, UAE University, Al Ain, UAE

**Keywords:** Climate change, Natural hazards

## Abstract

Heatwaves have afflicted human health, ecosystem, and socioeconomy and are expected to intensify under warming climate. However, few efforts have been directed to moist heat stress (MHS) considering relative humidity and wind speed, and moist heat stress risk (MHSR) considering exposure and vulnerability. Here we showed MHS and MHSR variations across China during 1998–2100 using China Meteorological Administration Land Data Assimilation System datasets, the 6th Coupled Model Intercomparison Project (CMIP6) merged datasets, Gross Domestic Product, population and leaf area index. We detected increased MHS across China under different Shared Socioeconomic Pathways (SSPs). Specifically, the historical MHS occurred mostly during mid-July to mid-August. We found increasing trends of 0.08%/year, 0.249%/year, and 0.669%/year in the MHS-affected areas under SSP126, SSP245, and SSP585, respectively. Furthermore, we observed the highest increasing rate of MHSR in Northwest and Southwest China, while the MHSR across Northeast and North China under SSP126 shifted from increasing to decreasing trends. Noteworthy is that the increasing trend of MHSR under SSP585 is 1.5–2.6 times larger than that under SSP245, especially in North and South China. This study highlights spatiotemporal evolutions of MHS and MHSR and mitigation to moisture heat stress in a warming climate.

## Introduction

Recent decades have witnessed amplifying air temperature in the backdrop of warming climate^1^, exerting negative impacts on human health, ecosystem and socioeconomy^2–4^. China, the largest developing country with the largest population and booming socioeconomy in the world, is undergoing amplifying heatwaves due to climate changes and human activities, such as urbanization^5–8^. Thorough investigation of spatiotemporal patterns and future evolutions of heat stress is the first step into preparedness and mitigation to heat-related disasters^9,10^. This is the motivation of this current study.

Heatwaves have direct impacts on human health such as heatwave-related diseases and mortality^11^. However, the heatwave has various concepts with emphases on specific aspects of heatwaves and most heatwaves were defined by air temperature only^12–14^. Relative humidity (RH) and wind speed (WS) are also critical in heatwave processes, and RH and WS can substantially modulate impacts of heatwaves on thermoregulation^15,16^. Unfortunately, few studies have introduced these two variables into the analysis of heatwaves^9,16,17^.

So far, few efforts have been directed to moist heat stress risk (MHSR) considering MHS (moist heat stress), population, GDP, and vegetation cover across China, though MHS has disastrous effects on human health as well as socioeconomy^18,19^, and particularly the exposure and vulnerability under MHS^20–22^. A bunch of studies analyzed differences amongst people given impacts by high temperature from viewpoints of population density, gender, age, education, income, housing quality, pre-existing medical conditions, minority status, poverty, social networks, home amenities (e.g., air conditioning)^19,21,23,24^, implying significance of exposure to MHS. Besides, effects of heatwaves can also be alleviated by some factors such as vegetation cover, surface albedo, irrigation, economic development, medical conditions, and warning levels^7,8,25–27^. Therefore, more variables than ever have been studied should be introduced into analysis of MHS. In this case, we attempted to evaluate MHSR across China since that China has various climatic zones with different population densities. Systematic evaluations of MHSR across China can greatly help to enhance mitigation to MHS at regional scales. This point justify the significance of this current study.

The objectives of this study therefore are to: (1) realize the data fusion by fully considering the systematic error of the CMIP6 model to improve its modelling accuracy; (2) delineate spatiotemporal evolutions of MHS considering the influences of RH and WS over China; and (3) quantify the spatiotemporal evolutions of regional MHSR across China considering population, GDP and leaf area index (LAI). The study seeks to foster improved understanding of MHS evolution and enhancement of mitigation of MHSR in a warming climate.

## Results and discussion

### Evaluation of CLDAS and CMIP6 datasets

**Figure 1:**
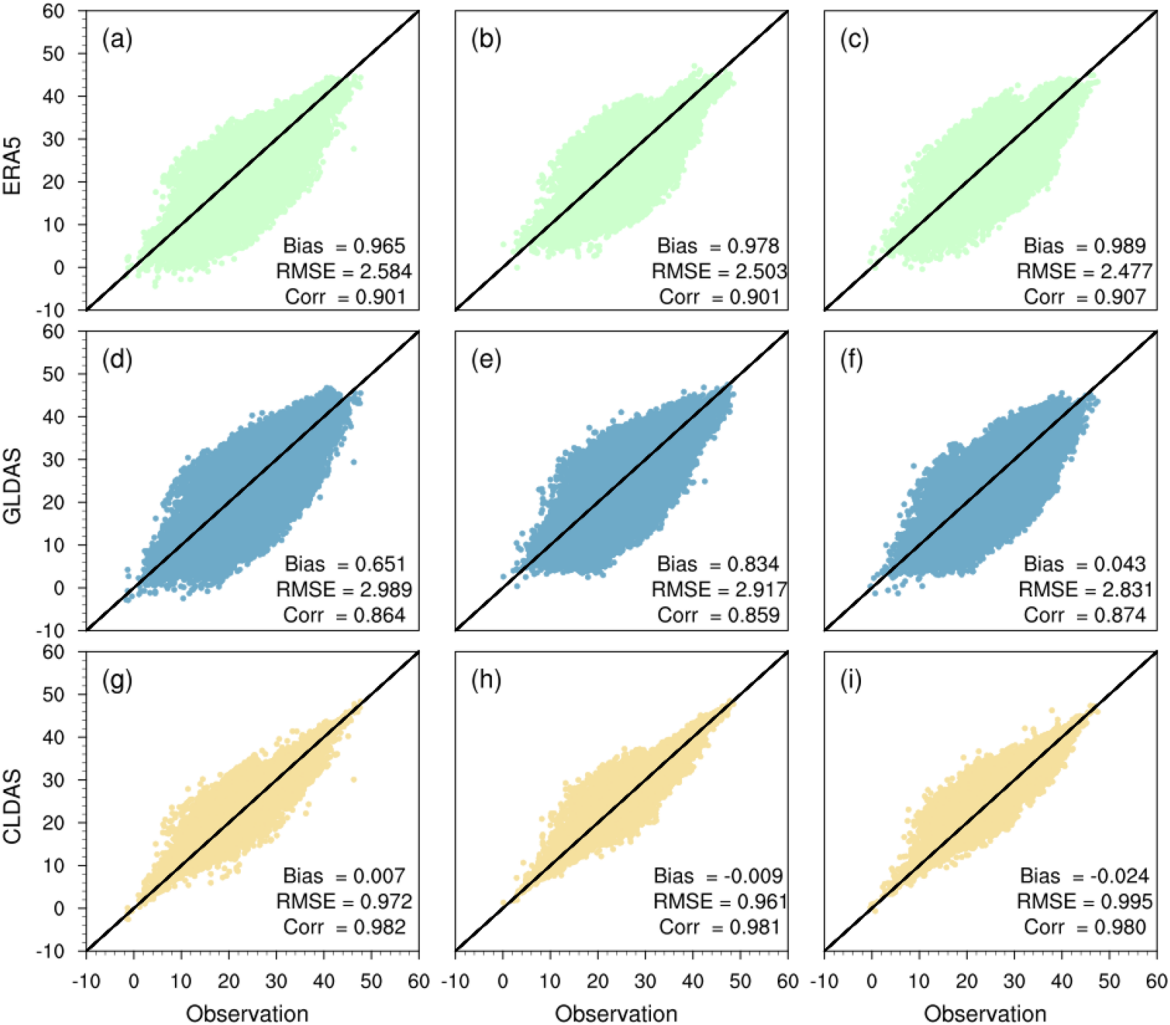
Performance evaluation of ERA5, GLDAS, and CLDAS in describing the daily maximum temperature changes during June, July, and August from 2008 to 2017 based on bias, RMSE, and correlation analysis. The first column of subplots shows the evaluation results for June, and the second and the third column are for July and August.

To ensure calculation accuracy of humidity (HI) and Steadman Index (SI), we evaluated the quality of the CLDAS data using the observed data of CMA (The distribution of observation stations can be seen in Fig. S1) and compared the CLDAS with the Global Land Data Assimilation System dataset (GLDAS)^28^ and the fifth-generation ECMWF reanalysis datasets (ERA5)^29^ The bias, RMSE, and Corr. of ERA5, GLDAS, and CLDAS with in-situ observed daily maximum temperature are shown in Fig. 1. In general, the bias of CLDAS daily maximum temperature was smaller than that of ERA5 and GLDAS daily maximum temperature. The RMSE of CLDAS was 0.9 °C, which was distinctly smaller than ERA5 (~ 2.5 °C) and GLDAS (~ 2.9 °C), and the Corr. between CLDAS temperature data and the in-situ observed temperature was about 0.98, being significantly larger than ERA5 (~ 0.9) and GLDAS (~ 0.86). The evaluation results for the specific humidity and wind speed can be found in Fig. S5 and Fig. S6 of the Supplementary Information. Therefore, the CLDAS data performed better than ERA5 and GLDAS data in describing changes in the summer daily maximum temperature and the specific humidity across China. Yin et al. used data such as air temperature fused with station observations to analyze apparent temperature in the one belt and one road region, whose the RMSE of air temperature is between 1 and 2 °C^16^, which indirectly verified the accuracy of our data source.

**Figure 2:**
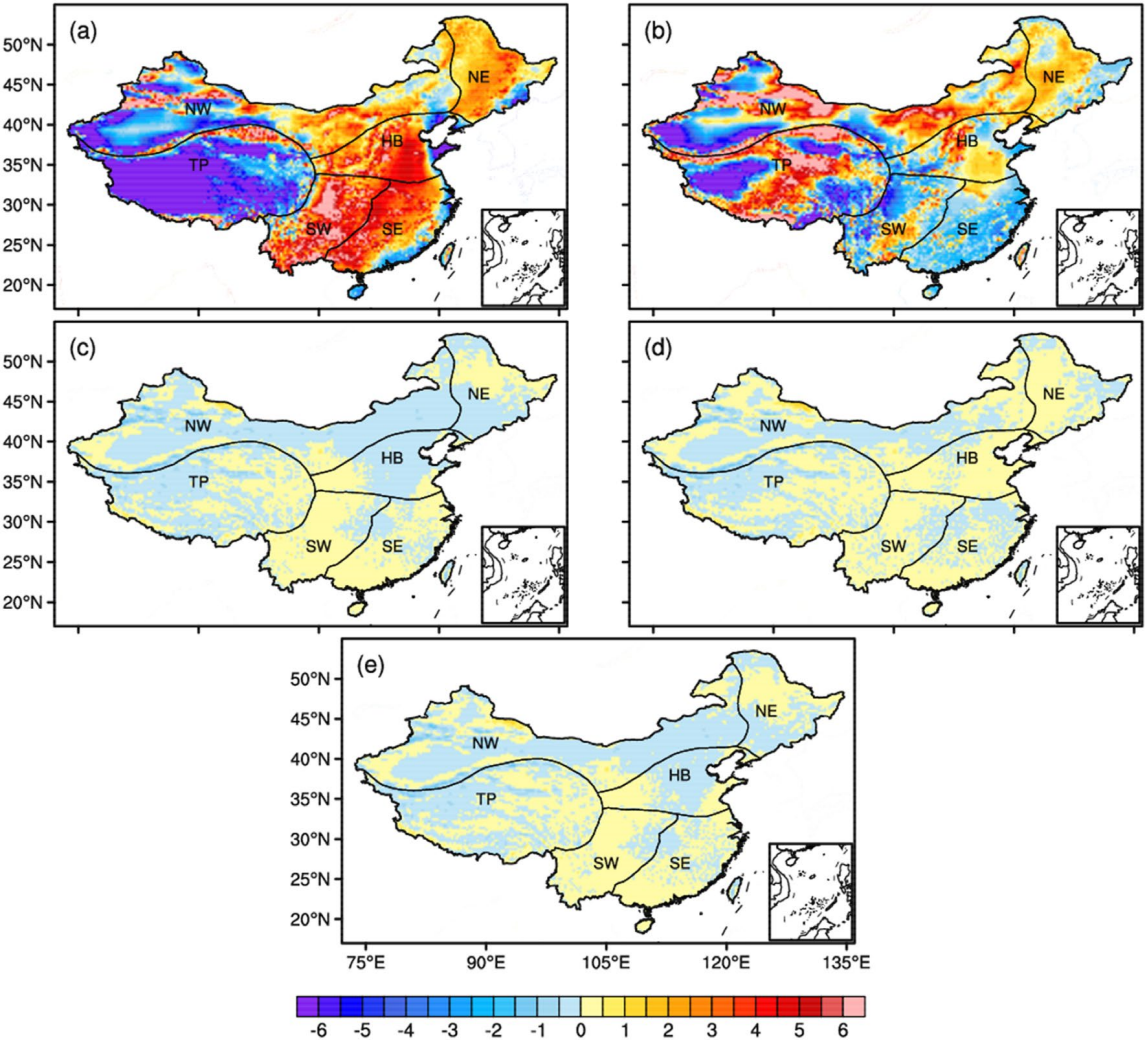
The temperature bias of the original data, the SSD-based processed data, and model-based fusioned data (unit:  °C). (**a**) original FGOALS outputs; (**b**) original CanESM outputs; (**c**) SSD-based processed FGOALS outputs; (**d**) SSD-based processed CanESM outputs; and (**e**) model-fusion outputs. The NE, HB, SE, NW, SW and TP represent the North East, North China, South East, North West and South West of China, the Tibetan Plateau respectively. The figure was created using the NCAR Command Language 6.4.0 (https://www.ncl.ucar.edu).

To ensure the accuracy of the CMIP6 data after the spatial downscaling and data fusion. Here we calculated the bias between CLDAS and the original FGOALS and CanESM outputs, the SSD-based processed and the fusioned FGOALS and CanESM outputs during the period of 1998 to 2019 (Fig. 2). We found a large positive bias (~ 2–6 °C) between the original FGOALS data and the CLDAS in Eastern China, and a large negative bias (− 6 to − 2 °C) in West China (Fig. 2a). We aslo detected a negative bias (− 3 to − 1 °C) between CanESM outputs and CLDAS in South China (Fig. 2b), a positive bias (2–3 °C) in most parts of North and Northeast China, a larger positive bias in the middle of the Tibetan Plateau (4 to 6 °C), and a large negative bias in the western part of the Tibetan Plateau (− 6 to − 2 °C). The bias of the FGOALS and CanESM was reduced using the SSD method, and the bias was − 1 to 1 °C (Fig. 2c,d). The TC-based on fusion determines the weight of the models based on the covariance of each model. Here we also included advantages of FGOALS and CanESM data after SSD-based data processing. The bias was small in some regions such as North China and Northeast China. The bias of relative humidity can be found in Fig. S7 of the Supplementary Information, and the effect was the same as for air temperature, which was better than the original data by SSD-based and TC-based data processing. So we can see the systematic error of the CMIP6 datasets in different regions calculated by the TC method based on this systematic error for CMIP6 data fusion, where the bias of temperature is between − 1 to 1 °C. While Sun et al. calculated an arithmetic average of the CMIP6 models to analyze the heat extreme intensity in Southeast Asia, which may have some limitations on the threshold estimation of heat extreme intensity. For example, CanESM5 temperatures are systematically high and are not error-corrected, manifesting an earlier emergence of the global warming thresholds than other models.

### Tendency of MHS in China

**Figure 3:**
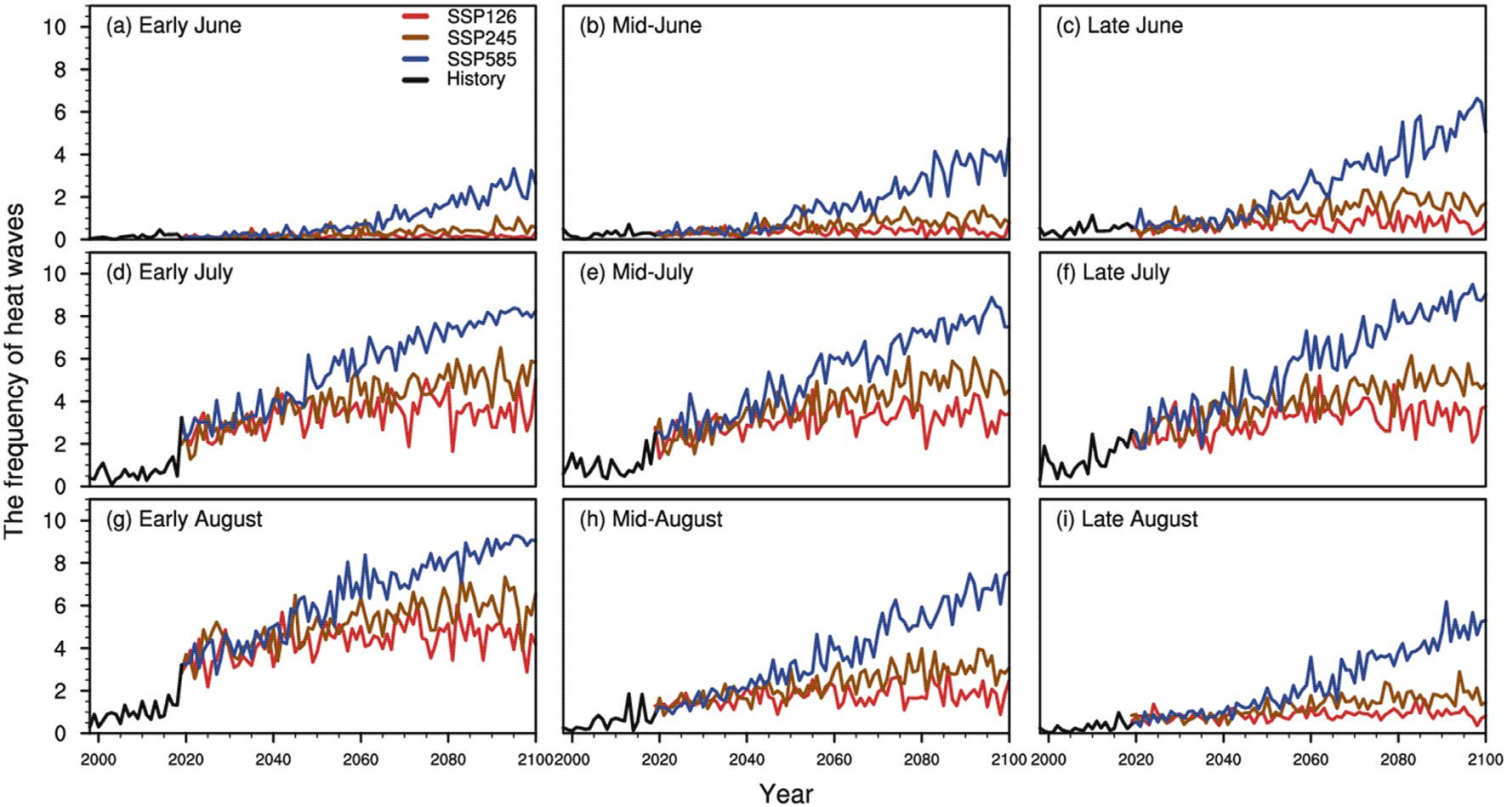
Temporal evolution of summer HI MHS frequency under different scenarios in the early, middle, late of each month from 1998 to 2100. (**a**–**c**) the early, middle, late of June; (**d**–**f**) the early, middle, late of July, and (**g**–**i**) the early, middle, late of August. The solid black curves show a historical trend of the MHS-affected area. Red/brown/blue curves denote the future trend of the MHS-affected areas under SSP126, SSP245, SSP585 scenarios.

From changes in monthly summer HI under different scenarios from 1998 to 2100 (Fig. 3, Table 1), we found that MHS mainly occurred from early July to early August. Besides, increased MHS frequency was followed by moderate changes under SSP126 and SSP245 scenarios. Specifically, the increasing magnitude of MHS frequency under SSP245 was larger than that under SSP126, while the increasing magnitude of MHS frequency under SSP585 was larger than that under SSP126 and SSP245. In a temporal sense, the MHS frequency under SSP126 and SSP245 was relatively low in early June, mid-June, and late June (Fig. 3a–c), while the MHS frequency was subject to a gradual increase under SSP585 and the MHS frequency was up to 6 times larger in late June. In July (Fig. 3d–f), the MHS frequency under SSP126 was subject to increase and was followed by moderate changes near 2060. Comparatively, the MHS frequency rapidly increased under SSP245 and then slowed down near 2060. The MHS frequency under SSP585 had an increasing tendency with an increasing rate of 0.0736/year in early July, 0.0743/year in mid-July, and 0.0853/year in late July. For MHS changes in August (Fig. 3g–i), the MHS frequency had an increasing tendency under SSP126 and had moderate changes near 2060. The MHS frequency had a rapid increasing tendency under SSP245 and the increasing tendency slowed down near 2060. Specifically, the MHS frequency increased under SSP585 with 0.075/year with an increasing magnitude in early August, 0.0786/year in mid-August, and 0.063/year in late August. The frequency of SI MHS (Fig. S8) changed following similar changing patterns when compared to the changes of HI MHS. Hence, MHS occurred mainly during July to early August. Besides, a higher MHS frequency was found under the SSP585 scenario when compared to that under SSP126 and SSP245 scenarios. These findings had profound implications for mitigation to MHS hazards in a warming climate and how to adapt human behavior to climate changes.

**Table 1:** Trends of summer HI MHS frequency under different scenarios in the early, middle, late of each month.

	Early June	Mid-June	Late June	Early July	Mid-July	Late July	Early August	Mid-August	Late August
SSP126	0.000	0.002	0.003	0.014	0.014	0.017	0.017	0.007	0.004
SSP245	0.006	0.010	0.020	0.038	0.038	0.034	0.032	0.024	0.016
SSP585	0.033	0.048	0.068	0.074	0.074	0.085	0.075	0.079	0.063

**Figure 4:**
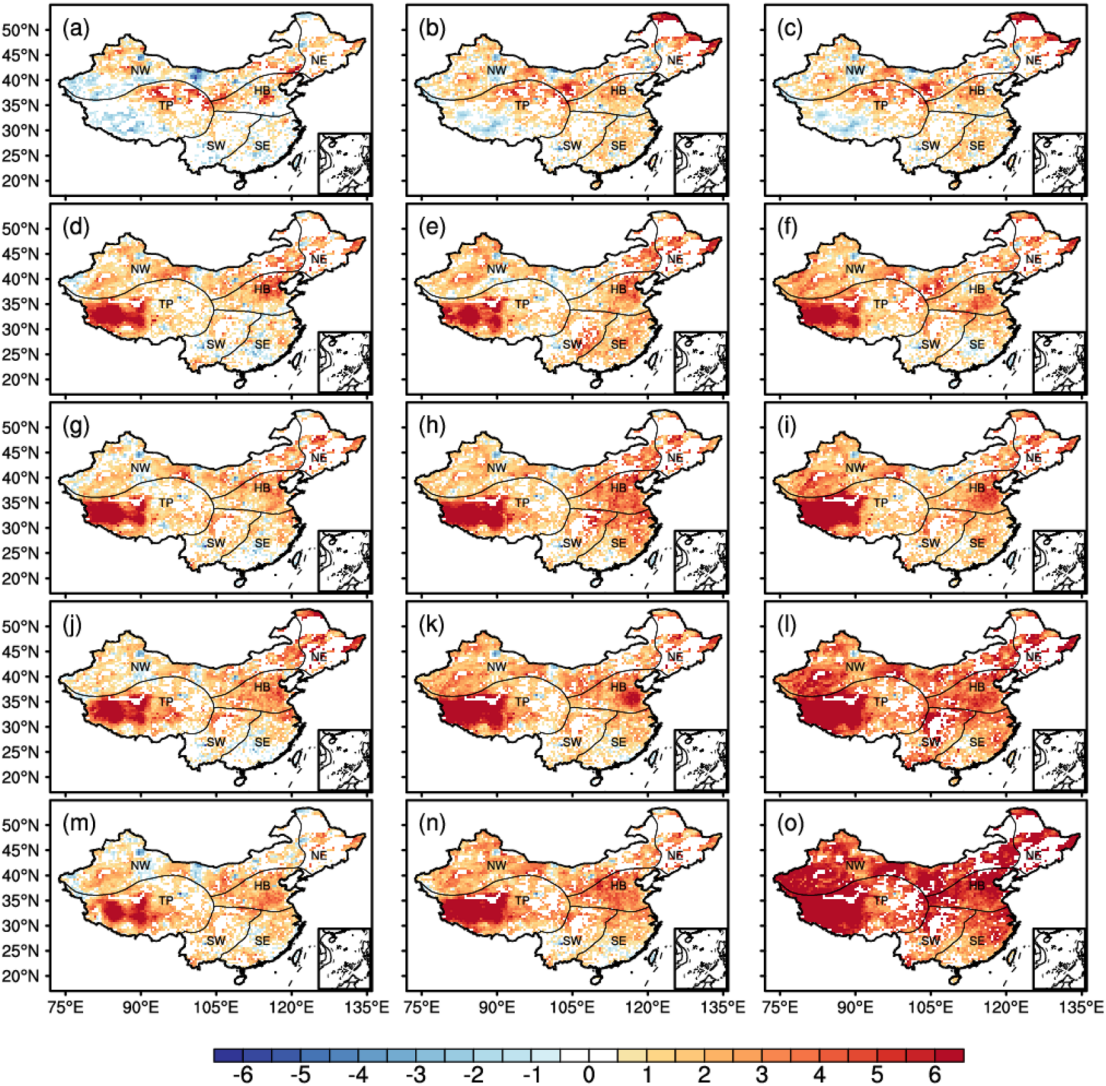
Spatial evolution of the HI MHS intensity by the CEEMDAN method. (**a**–**c**) show HI MHS intensity during 2000, 2010, and 2020. (**d**–**f**) show HI MHS intensity during 2040 (**d**–**f**), 2060 (**g**–**i**), 2080 (**j**–**l**), 2100 (**m**–**o**) respectively. Column from left column to the right shows HI MHS intensity under SSP126, SSP245, SSP585. The NE, HB, SE, NW, SW and TP represent the North East, North China, South East, North West and South West of China, the Tibetan Plateau respectively. The figure was created using the NCAR Command Language 6.4.0 (https://www.ncl.ucar.edu).

The spatial evolution of HI MHS intensity in 2000, 2010, 2020, 2040, 2060, 2080, and 2100 based on the CEEMDAN method (Fig. 4) showed that HI MHS intensity during 2000 (Fig. 4a), 2010 (Fig. 4b), and 2020 (Fig. 4c) increased in the Tibetan Plateau, the eastern part of Xinjiang, and the southeastern part of Yunnan Province. In 2040 (Fig. 4d–f), increased HI MHS intensity was found in North China, Northwest China, and the Tibetan Plateau under SSP126, SSP245 and SSP585 scenarios. The highest HI MHS intensity was observed in the Tibetan Plateau and was followed by the HI MHS intensity in North China and Northeast China. Decreased HI MHS intensity was depicted in South China under the SSP126 and SSP585 scenarios. In 2060 (Fig. 4g–i), the HI MHS intensity would increase under the SSP126, SSP245 and SSP585 scenarios in North China, Northeast China, and the Tibetan Plateau, and it is particularly the case for HI MHS intensity in North China. As for MHS intensity changes in South China, the MHS intensity and the spatial extent of under SSP126 was lower than that under SSP245; and MHS intensity under SSP245 was lower than that under SSP585, while the MHS intensity under SSP585 was higher than that in 2060 in North China, central China, and the middle and lower Yangtze River. In 2100 (Fig. 4m–o), the MHS intensity under SSP126 and SSP245 was similar to that in 2080 (Fig. 4j–l). Besides, we found decreasing MHS intensity in the Tibetan Plateau under SSP126, while the MHS intensity in most areas of China would increase significantly by 2100 under SSP585, especially in North China, Northeast China, and the Tibetan Plateau.

The spatial evolution of the SI MHS intensity considering wind speed indicates that the changing magnitude of SI MHS intensity was lower than HI MHS intensity (Fig. S11). Under SSP126 and SSP245 scenarios, the SI heatwave intensity in the Tibetan Plateau, North China, and Northeast China evidently increased and then followed by moderate changes. The SI heatwave intensity in China would generally increase under the SSP585 scenario and the largest increasing magnitude of SI heatwave intensity was found in the Tibetan Plateau, North China, and Northeast China. Little changes in SI heatwave intensity were observed in South China.

These results indicated increased frequency (Fig. S9 and S10), intensity (Fig. S11), duration (Fig. S12 and S13), and heatwave-affected area (Fig. S14) of MHS in China in the backdrop of global warming, especially under the SSP585. Specifically, a large increase of MHS was found in the the Tibetan Plateau, which is consistent with the related studies^16,30^. This indicates the sensitivity of the Tibetan Plateau to climate change and the increase of MHS on the Tibetan Plateau would have an impact on the climate of China and other regions of the world through thermal and dynamical factors.

### Tendency of MHSR in China

**Figure 5:**
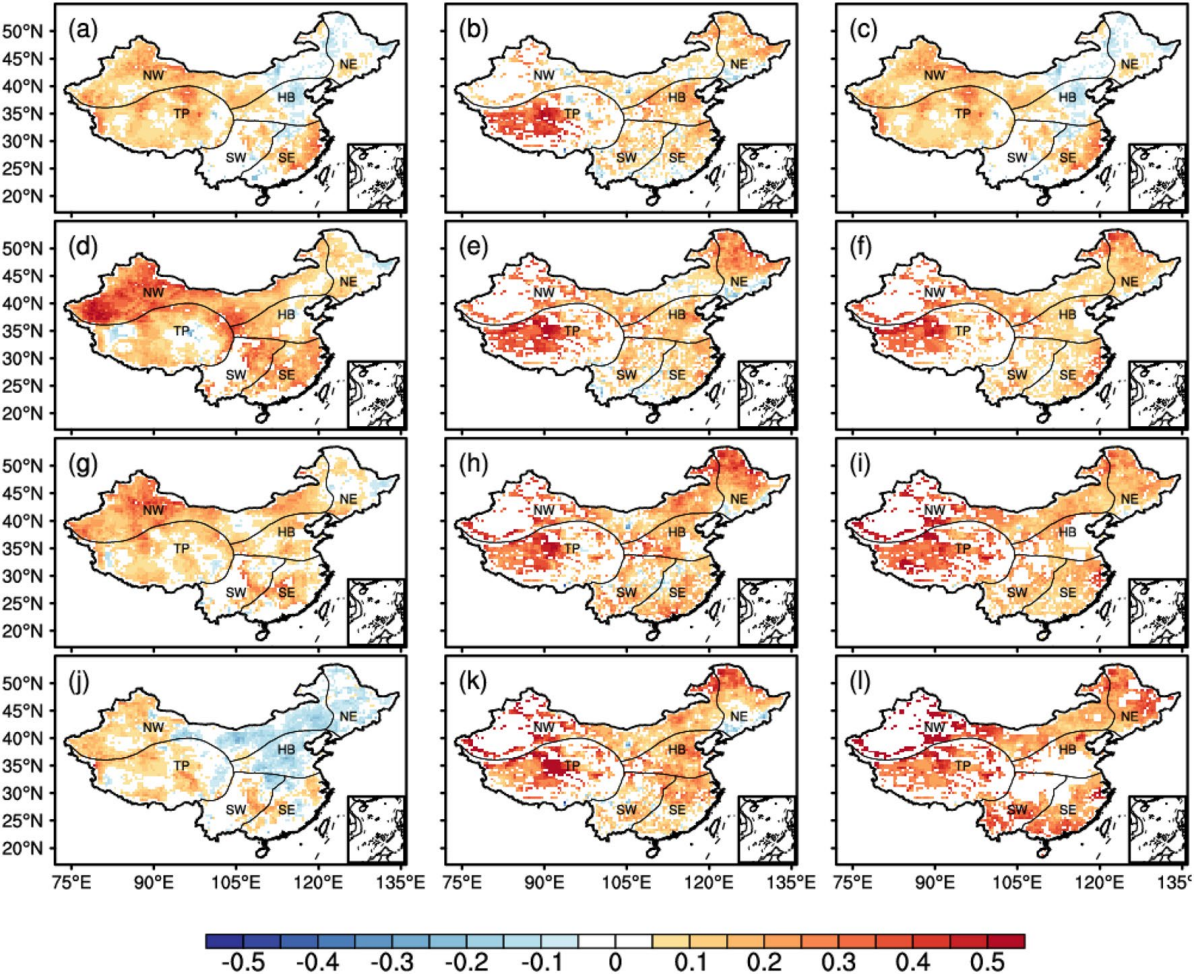
Spatial evolution of the MHSR by the CEEMDAN method. (**a**–**c**) show HI heatwave intensity during 2040. (**d**–**f**) show MHSR during 2060 (**d**–**f**), 2080 (**g**–**i**), 2100 (**j**–**l**) respectively. Column from the left column to the right shows HI heatwave intensity under SSP126, SSP245, SSP585. The NE, HB, SE, NW, SW and TP represent the North East, North China, South East, North West and South West of China, the Tibetan Plateau respectively. The figure was created using the NCAR Command Language 6.4.0 (https://www.ncl.ucar.edu).

We calculated the spatial evolution of MHSR in 2040, 2060, 2080, and 2100 based on 2020 using the CEEMDAN method (Fig. 5). From different SSP scenarios in the same year, such as in 2040 (Fig. 5a–c), increased MHSR was found in the southern region of Northeast China, the western region of North China, the central-eastern region of South China, the northern region of Southwest China, the western region of Northwest and the Tibetan Plateau under SSP126, and the increase in MHSR was most pronounced in the central-eastern region of South China. The MHSR increases in most regions of Northeast and North China, north-central of South China, and most regions of Southwest China under SSP245, but is less pronounced in Northwest China. The MHSR increases in the western region of Northern China, the east-central region of South China, most regions of northwest China, and the Tibetan Plateau under SSP585, and is most pronounced in the east-central part of South China. In 2060 (Fig. 5d–f), increased MHSR was found in the most regions of Northeast China, the western region of North China, most region of South China, the east-central region of Southwest China, the central region of Northwest China and the Tibetan Plateau under SSP126, and the increase was most pronounced in Northwest China, east-central region of Southwest China and most region of South China. The MHSR was found in most region of Northeast China, the west-central region of North China, the east-central region of South China, the central region of Southwest China, the central region of Northwest, and the western region of the Tibetan Plateau under SSP245 under SSP585. In 2080 (Fig. 5g–i), increased MHSR was found in the south-central region of North China, the central region of South China, the eastern region of Southwest, the central region of Northwest, and Tibetan Plateau under SSP126. And the MHSR in the northern region of Northeast China, the west-central region of North China, most regions of South China and Southwest China, the east-central region of Northwest China and the western region of Tibetan Plateau under SSP245 under SSP585 are also increasing accordingly. In 2100 (Fig. 5j–l), increased MHSR was found in northeastern and the southwestern region of South China, the east-central region of Southwest China, the northwest region of Xinjiang, and most regions of the Tibetan Plateau under SSP126, in contrast to decreasing MHSR in North China, Northeast China, and the east-central region of Northwest China. The MHSR under SSP245 in the northern part of Northeast China, most of Northwest China, the north-central region of South China, most regions of southwest China, the east-central region of Northwest China, and the western region of Tibetan Plateau are increasing accordingly. In contrast, the MHSR under SSP585 increases in most regions of Northeast China, the northern region of North China, the southern region of South China, the southern region of Southwest China, the central-eastern region of Northwest China, and the western region of the Tibetan Plateau.

From the same SSP in different years, for the SSP126 scenarios (Fig. 5a, d, g, j), the magnitude and extent of MHSR showed a trend of increasing and then decreasing in different regions of China, especially MHSR decreased in Northeast China, North China and the east-central region of Northwest China in 2100. For SSP245 scenarios (Fig. 5b, e, h, k), the MHSR in Northeast China firstly shows an increase and then tends to stabilize, especially in the northern region of Northeast China, and the MHSR in North China further expands in scope and magnitude, the South China shows an increase in scope and magnitude and then gradually concentrates in the northern region of South China, while the Northwest China, Southwest China and the Tibetan Plateau show an increase and then tends to stabilize. For the SSP585 scenarios (Fig. 5c, f, i, l), the extent and magnitude of MHSR in Northeast China first decreased and then increased, the MHSR in North China showed a trend of expanding from the west of North China to the north of North China, and the extent and magnitude of MHSR in South China and Southwest China showed a trend of first increasing and then gradually expanding to the southern region of South China and southern regions of Southwest China. The MHSR in Northwest China and the Tibetan Plateau show a trend of increasing firstly and then tending to be unchanged.

We integrated the exposure of MHS, population, GDP and LAI under different scenarios to represent the spatiotemporal evolutions of MHSR. Yin et al. analyzed the spatiotemporal distribution and risk of heat waves by apparent temperature, population and NDVI data, and the results showed that the eastern part of China deepens economic development while taking heat wave risk into account^31^. Here we better reflected the spatial variations by CEEMDAN method under different scenarios. While GDP is also an important factor in heat stress risk for low-income people. There are relatively few countermeasures for heat stress, for example, expensive air conditioners are a burden for them^32^, and the level of economic development in North China is relatively low compared to Southeast China. Therefore it can be seen that the spatial extent of MHSR in North China is wider compared to Southeast China.

**Figure 6:**
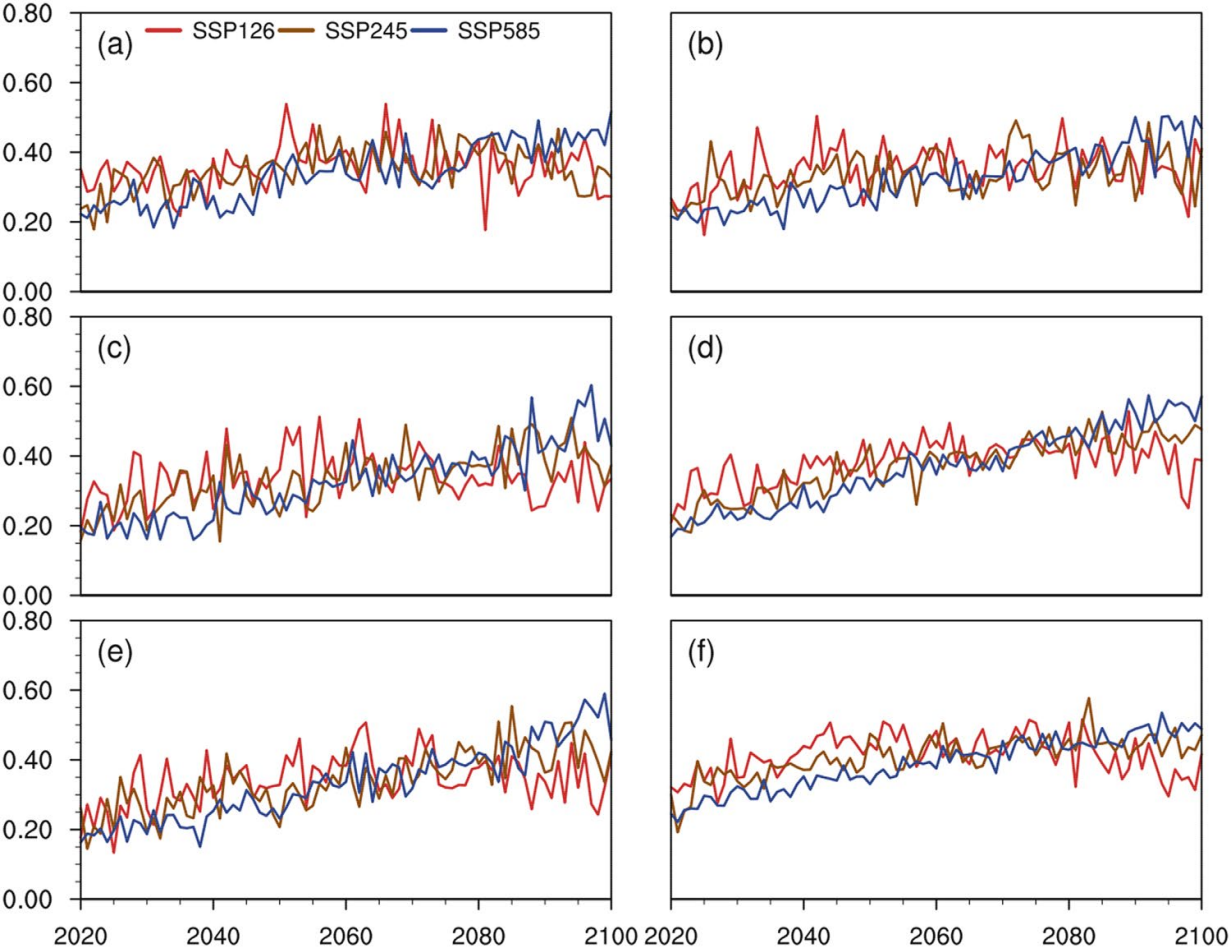
Temporal evolution of MHSR of different regions of China under different scenarios from 2020 to 2100. (**a**) Northeast China; (**b**) North China; (**c**) South China; (**d**) Northwest China; (**e**) Southwest China; (**f**) the Tibetan Plateau. Red/brown/blue curves denote the future trend of the MHSR under SSP126, SSP245, SSP585 scenarios.

To further analyze the temporal trends of MHSR, we analyzed the temporal changes of MHSR in different regions in China using the MK test. For Northeast China (Fig. 6a), MHSR first increased and then showed a decreasing trend under SSP126, with an overall increase of 0.00049/year, while MHSR showed a continuously increasing trend under SSP245 and SSP585, with 0.00108/year and 0.00308/year, respectively. For North China (Fig. 6b), the MHSR slowly increased under SSP126, with an overall increase of 0.00049/year, and the MHSR also showed a continuous increase under SSP245 and SSP585, with 0.00108/year and 0.00308/year, respectively. For South China (Fig. 6c), the MHSR first increased and then stabilized under SSP126, with an overall increase of 0.00037/year, while the MHSR showed a continuous increase under SSP245 and SSP585, with 0.00233/year and 0.00385/year, respectively. For Northwest China (Fig. 6d), the MHSR increased and then decreased under SSP126, with an overall increase of 0.00145/year, while the MHSR showed a continuous increase under SSP245 and SSP585, with 0.00308/year and 0.00462/year, respectively. For Southwest China (Fig. 6e), MHSR increased and then decreased under SSP126, with an overall increase of 0.001408/year, while MHSR showed a continuous increasing trend under SSP245 and SSP585, with 0.00282/year and 0.00423/year, respectively. For the Tibetan Plateau (Fig. 6f), MHSR increased and then decreased under SSP126, with an overall increase of 0.00032/year, while MHSR showed a continuously increasing trend under SSP245 and SSP585, with 0.00166/year and 0.00305/year, respectively. Looking at the same SSP in different subregions, the MHSR increases the most in Northwest China and Southwest China, followed by South China and North China, and Northeast China and the Tibetan Plateau had relatively small increased in MHSR because of their latitudinal position and altitude. Besides, the increasing trend of MHSR under SSP585 is 1.5–2.6 times that of SSP245, especially in North China.

## Discussion

In the context of global warming, the burning of fossil fuels, deforestation, rapid urbanization, growing population brought about by economic development and the anthropogenic heat generated by the overuse of energy, people will face more and more MHS in the future, especially for China, a developing country with a huge population^10,11^. The frequency of moist heat stress events, increased population exposure, and increasing human health problems caused by MHS have affected economic development to some extent, especially for urban residents with poor ventilation, buildings and impervious surfaces that absorb and retain heat, as well as increased high humidity under anthropogenic heat release^16,24,25^.

Nowadays, more and more countries are taking corresponding measures to reduce the MHSR, such as urban greening, irrigation, central air conditioning and the construction of corresponding heat avoidance facilities^7,21,27^. Urban greening increases the leaf area index, absorbing more solar radiation and reducing the temperature increase due to absorption of solar radiation on the ground. While the transpiration of vegetation can effectively improve the heat-sensing and latent heat changes in the subsurface, regulating the relative humidity and reducing heat stress to a certain extent. Development of socioeconomy can allow air conditioning and which can greatly alleviate indoor heat stress-induced healthy issues. While, energy consumption also generates myriad anthropogenic heat^22,23,27^. Here we found changing pattern of MHSR as “increases-decreases” under SSP126 scenarios in most regions, while for SSP245 and SSP585 scenarios, the increasing trend of MHSR under SSP585 scenarios is 1.5–2.6 times larger than that of SSP245 scenarios. So the sustainable development path can help to reduce the MHSR such as the carbon neutrality and carbon peaking policies currently being implemented in China.

In terms of data source uncertainty, the CLDAS air temperature, relative humidity and wind speed fused with more than 50,000 in situ observations from the China Meteorological Administration (CMA) have a higher accuracy than ERA5 and GLDAS. However, sparse distribution of CMA station observations in western China when compared to the eastern China results in relatively lower quality of CLDAS data in the western than in the eastern China^33^. Besides, we spatially downscaled and fused the CMIP6 data, uncertainty is still unavoidable in the CMIP6 data under different climate models due to different model types, parameterization schemes, and spatial resolutions^14,31^. What's more, the GLASS leaf area index data we used from 1998–2018 are mainly based on the results of satellite inversion, which have relatively higher accuracy and are better than MODIS and AVHRR. Certain errors can also be expected for areas with remarkable heterogeneous vegetation cover^34,35^. The CMIP6 data during 2020–2100 used for the LAI data under different scenarios are mainly generated by climate models, which have higher quality than the CMIP5 model, but there is an overestimation of LAI in non-forested regions^36^ .

## Conclusions

In this study, we depicted MHS and MHSR changes in both space and time across China during a period of 1998–2100 based on CLDAS datasets, CMIP6 datasets, GDP, population and LAI datasets under SSP126, SSP245, and SSP585 scenarios. We have obtained the following important and interesting conclusions:

Considering the coarse spatial resolution and systematic errors of the models in CMIP6, we adopt the SD and TC methods to realize the spatial downscaling of the two models and the calculation of systematic errors for each grid point of the models, respectively. Then we calculate their weights according to the model errors and realize the fusion of CMIP6 model data, so as to reduce the uncertainty brought by the data source and ensure the accuracy and quality of the data source. It can be found that the fusion results are significantly better than the original data, especially the bias was obviously reduced.

The MHS in China showed an increasing temporal trend in a warmer climate. Firstly, the MHS-affected area in China has gradually increased, with an average annual increase of 0.399% and 0.479% under HI and SI from 1998 to 2020. The MHS-affected area under SSP126 first increased and then stabilized from 2021 to 2100. The MHS under SSP245 and SSP585 were gradually increasing, and the MHS-affected area under SSP585 was higher than that under SSP126 and SSP245. Besides, the frequency of historical MHS mainly occurred from mid-July to mid-August. The MHS under SSP126 also mainly occurred from early July to early August, while the MHS under SSP245 and SSP585 would continue from early July to the end of August. Meanwhile, the frequency under SSP585 was higher than that under SSP126 and SSP245.

The MHSR in China showed an increasing temporal trend in a warmer climate except for Northeast China and North China under SSP126. Firstly, it can be seen from the spatial evolution that the extent and magnitude of MHSR in Northeast China, North China, and the eastern regions of Northwest China showed an increasing trend before decreasing, and the extent of MHSR in South China and Southwest China increased before decreasing under SSP126. And the range of MHSR under SSP245 in most regions of China showed an increasing trend, but the magnitude was relatively larger than SSP126 and smaller than SSP585. Besides, the range of MHSR under SSP585 decreases in some regions, but the magnitude increases more, such as the center of large values of MHSR in North China gradually shifts to the northern regions (Beijing-Tianjin-Hebei region), and the center of large values of MHSR in South China gradually shifts to the southern regions. In terms of time evolution, MHSR increases and then decreases or smoothly under SSP126 in most regions, while for SSP245 and SSP585, the increasing trend of MHSR under SSP585 is 1.5–2.6 times that of SSP245, especially in North China.

All in all, we analyzed the intensity, affected area, duration, and frequency of MHS in China, and explored the spatial and temporal evolution of MHSR in different regions of China considering the hazards, exposures, and vulnerability by combining population, GDP, and LAI data. These results can provide a basis for the management and response to moist heat stress risk in different regions.

## Method

### The spatial downscaling and data fusion of the CMIP6 datasets

To ensure the accuracy of the CMIP6 data used, we used the spatial disaggregation (SD) statistical downscaling method for spatial downscaling of the CMIP6 data^37^. Considering that the bias in monthly average temperature by the traditional SD method would smooth information, especially the temperature, we improved a sliding SD method (SSD) at a three-day scale (Fig.S2). We also fully considered and calculated the error of the model using the Triple Collocation method (TC)^38^. Then we calculated the weights of model according to the error of model and obtained the fusion of FGOALS and CanESM models.

The variance and covariance formulas for CLDAS, FGOALS and CanESM models are shown as:

$$ \sigma^{2}_{i} = \beta^{2}_{i} \sigma^{2}_{\theta } + \sigma^{2}_{{\varepsilon_{i} }} $$

1 $$ \sigma_{ij} = \beta_{i} \beta_{j} \sigma^{2}_{\theta } + \sigma_{{\varepsilon_{i} \varepsilon_{j} }} $$

The systematic error of the CLDAS, FGOALS and CanESM models data sets are as follows:

$$ \sigma^{2}_{rX} = \sigma^{2}_{X} - \frac{{(\sigma_{XY} - \sigma_{{\varepsilon_{x} \varepsilon_{y} }} )(\sigma_{XZ} - \sigma_{{\varepsilon_{x} \varepsilon_{z} }} )}}{{\left( {\sigma_{YZ} - \sigma_{{\varepsilon_{z} \varepsilon_{y} }} } \right)}} $$

$$ \sigma^{2}_{rY} = \sigma^{2}_{Y} - \frac{{(\sigma_{XY} - \sigma_{{\varepsilon_{x} \varepsilon_{y} }} )\left( {\sigma_{YZ} - \sigma_{{\varepsilon_{z} \varepsilon_{y} }} } \right)}}{{(\sigma_{XZ} - \sigma_{{\varepsilon_{x} \varepsilon_{z} }} )}} $$

2 $$ \sigma^{2}_{rz} = \sigma^{2}_{X} - \frac{{\left( {\sigma_{YZ} - \sigma_{{\varepsilon_{z} \varepsilon_{y} }} (\sigma_{XZ} - \sigma_{{\varepsilon_{x} \varepsilon_{z} }} } \right)}}{{(\sigma_{XY} - \sigma_{{\varepsilon_{x} \varepsilon_{y} }} )}} $$

The weights of each model were obtained according to the error of each model, and the weights were calculated as:

$$ \omega_{{{\text{FGOALS}}}} = \frac{{1/\sigma_{{r_{FGOALS} }}^{2} }}{{\frac{1}{{\sigma_{{r_{{CL{\text{DAS}}}} }}^{2} }} + \frac{1}{{\sigma_{{r_{FGOALS} }}^{2} }} + \frac{1}{{\sigma_{{r_{CanESM} }}^{2} }}}} $$

3 $$ \omega_{{{\text{CanESM}}}} = \frac{{1/\sigma_{{r_{CanESM} }}^{2} }}{{\frac{1}{{\sigma_{{r_{{CL{\text{DAS}}}} }}^{2} }} + \frac{1}{{\sigma_{{r_{FGOALS} }}^{2} }} + \frac{1}{{\sigma_{{r_{CanESM} }}^{2} }}}} $$

Multi-model fusion was performed according to the weights of different models of CMIP6, and the CMIP6 results after multi-model fusion can be obtained:

4 $$ CMIP6_{TC} = \omega_{FGOALS} CMIP6_{FGOALS} + \omega_{CanESM} CMIP6_{CanESM} $$

The spatial downscaling and model-fusion procedure was shown in Fig. S2 and the sample figure was in Fig. S3.

### The calculation of MHSR

According to the risk triangle evaluation theory, the calculation of MHSR mainly takes into account hazard, exposure and vulnerability^21^. Hazard mainly considers the frequency, duration and intensity of MHS, exposure mainly considers the impact of MHS on population and GDP, and vulnerability mainly considers the mitigation effect of higher GDP and vegetation cover on MHS. All data were normalized before the MHSR was calculated, and the MHSR equation is as follows.

5 $$ {\text{Hazard}} = \left( {Frequency + Duration + Intensity} \right)/3 $$

6 $$ {\text{Exposure}} = \left( {Population + GDP} \right)/2 $$

7 $$ {\text{Vulnerability}} = \left( {{\text{GDP}} + LAI} \right)/2 $$

8 $$ {\text{MHSR}} = \left( {{\text{Hazard}}*0.{46} + {\text{Exposure}}*0.{31} - {\text{Vulnerability}}*0.{23}} \right) $$

### The calculation of MHS indices and MHS thresholds

Here we used the HI and the SI to identify MHS across China. HI involves air temperature and RH and SI involves the air temperature, relative humidity, and WS. The HI has been widely used for the evaluation of indoor and outdoor thermal conditions and human comfort^26,39^. HI can be computed as:

9 $$ {\text{Humidex}} = {\text{T}} + 0.5555 \times \left( {{\text{P}} - 10} \right) $$

10 $$ {\text{P}} = {\text{RH}}/100 \times 6.105 \times e^{{\left( {17.27 \times T/\left( {273.7 + T} \right)} \right)}} $$

SI can be calculated as^40^:

11 $$ {\text{SI}} = 1.07 \times T + 0.2 \times P - 0.65 \times V - 2.7 $$

where *T* denotes the air temperature, $RH$ denotes the relative humidity, *P* is vapor pressure, and *V* denotes the wind speed.

Here we used relative and absolute temperature thresholds to identify MHS and 90% percentile was taken as the relative temperature threshold for MHS^13,41^. The MHS of 3 °C higher than the long-term mean MHS is taken as the absolute temperature threshold. Given that the relative MHS threshold is lower than the absolute MHS threshold, the absolute MHS threshold can be accepted to define heatwaves^16^.The spatial distribution of the thresholds of the MHS can be found in Fig. S4 in Supplementary Information.

### Spatial–temporally varying trend

The Mann–Kendall (MK) test was used to detect the trends in the meteorological serie^42^. The separate spatiotemporally-varying trend of the heatwave from 1998 to 2100 was diagnosed using the Complete Ensemble Empirical Mode Decomposition with Adaptive Noise Analysis method (CEEMDAN)^43^. We diagnosed the value increment of the CEEDMAN spatial trend at a given time from 1998 to 2100, and the reference time was 1998 year, which represented accumulated warming from 1998:

12 $$ {\text{H}}\left( {\text{t}} \right) = \mathop \sum \limits_{k = 1}^{k} IMF_{k} + R\left( t \right) $$

13 $$ Trend_{CEEDMAN} \left( t \right) = R\left( t \right) - R\left( {1998} \right) $$

$\mathrm{H}\left(\mathrm{t}\right)$ is the original signal, ${IMF}_{k}$ is the Intrinsic Mode Function, and $R\left(t\right)$ is the extracted trend.

## Supplementary Information

Supplementary Information.

## Data Availability

The China Meteorological Administration Land Data Assimilation System (CLDAS) air temperature, specific humidity, and wind data with daily time scale and 0.25° spatial resolution from 1998 to 2020 are freely available at http://data.cma.cn^44^. The Coupled Model Intercomparison Project datasets (CMIP6) air temperature, specific humidity, wind data and LAI data under SSP126, SSP245, and SSP585 scenarios from 2021 to 2100 are freely available at https://esgf-node.llnl.gov/search/cmip6/^45^. The population and GDP from 2000 to 2019 are freely available at https://www.resdc.cn. The population and GDP data from 2020 to 2100 under SSP126, SSP245, and SSP585 scenarios are freely available at https://geography.nuist.edu.cn^46^ LAI datasets used the Global Land Surface Satellite (GLASS) LAI product from 1998 to 2018 are freely available at http://glass-product.bnu.edu.cn/introduction/LAI.html^47^. The datasets used and analysed during the current study available from the corresponding author on reasonable request.
