# Supplementary material for: Increased moist heat stress risk across China under warming climate
Source: Sci Rep. 2022 Dec 29;12:22548. doi: 10.1038/s41598-022-27162-2 (PMC9800580; doi:10.1038/s41598-022-27162-2)
Supplement: Supplementary file 1 — Supplementary Information. [file 41598_2022_27162_MOESM1_ESM.docx]

Supplementary Information for

**Increased moist heat stress risk across China under warming climate**

Shuai Sun^1,2,3^, Qiang Zhang^4*^, Vijay P. Singh^5^, Chunxiang Shi^3^, Gang Wang^1,2^, Wenhuan Wu^1,2^, Zexi Shen^1,2^

^1^State Key Laboratory of Earth Surface Processes and Resource Ecology, Beijing Normal University, Beijing, China.^2^Faculty of Geographical Science, Beijing Normal University, Beijing, China. ^3^National Meteorological Information Center, China Meteorological Administration, Beijing, China. ^4^Advanced Interdisciplinary Institute of Environment and Ecology, Beijing Normal University, Zhuhai 519087, China; ^5^Department of Biological and Agricultural Engineering and Zachry Department of Civil & Environmental Engineering, Texas A&M University, College Station, Texas, USA; National Water and Energy Center, UAE University, Al Ain, UAE.

**Contents of this file**

Section 1 to Section 10

**Introduction**

Supplemental materials include the description of the evaluation result of CLDAS data and CMIP6 data used in analysis. The supplemental materials also include the description of the sliding spatial downscaling and data fusion. These indicate the data we used have high spatial resolution and high accuracy. In addition, there are the frequency, intensity, and duration of SI MHS.

Section 1. Stations of CMA used for evaluation


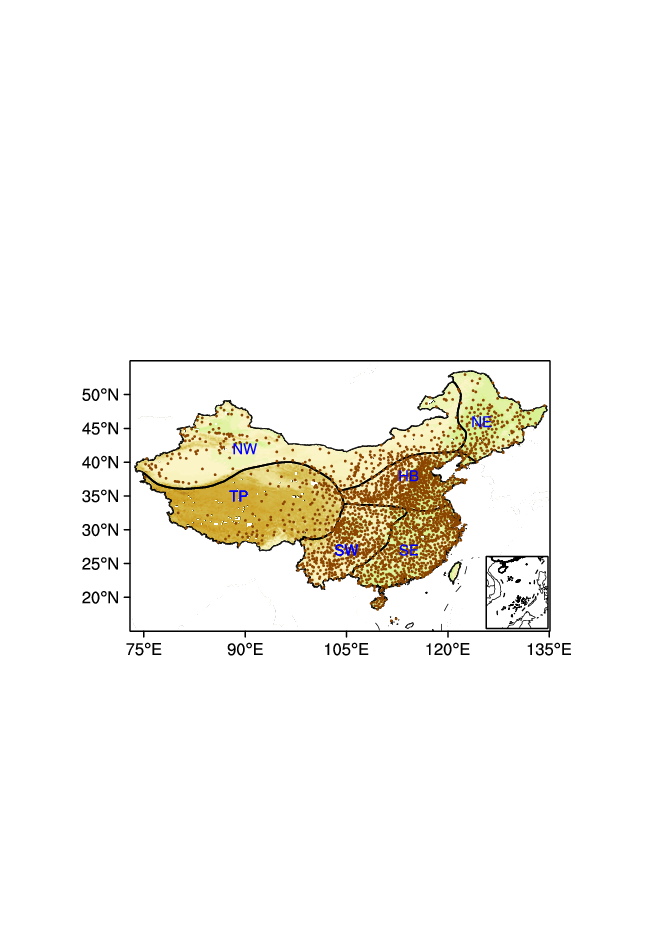


Fig. S1 Distribution of 2380 national temperature and relative humidity observation stations of CMA used for evaluation. The NE, HB, SE, NW, SW and TP represent the North East, North China, South East, North West and South West of China, the Tibetan Plateau respectively. The figure was created using the NCAR Command Language 6.4.0 (https://www.ncl.ucar.edu)

Section 2. The flowchart of the sliding SD and model fusion for CMIP6 different models

The detailed steps of the sliding SD method can be seen in Fig.S2. Firstly, interpolating the temperature, relative humidity, wind speed of FGOALS and CanESM historical data to 0.25°. Using the same period in the past 3 days as the sample, the deviations between the high-quality CLDAS and FGOALS and CanESM historical data were slidingly calculated. And apply the deviation of the temperature, relative humidity, and wind speed in the same time period to the FGOALS and CanESM in the scenarios, so as to achieve spatial downscaling.

The TC method is based on the following three assumptions: First, the errors of the three data sets are independent of each other. Second, the errors of the three data sets are independent of the true values. Third, the error is stable and does not change with time. Considering that different models in CMIP6 have certain systematic errors, the TC method was used to analyze the errors of the data under different scenarios of FGOALS and CanESM. The variance of historical data of CLDAS, FGOALS, and CanESM from 1998 to 2019 was calculated, based on the TC method. The weight of each model was determined according to the model variance, and the FGOALS and CanESM under different scenarios were merged to obtain the multi-model fusion results, and finally, HI and SI under different scenarios were calculated.


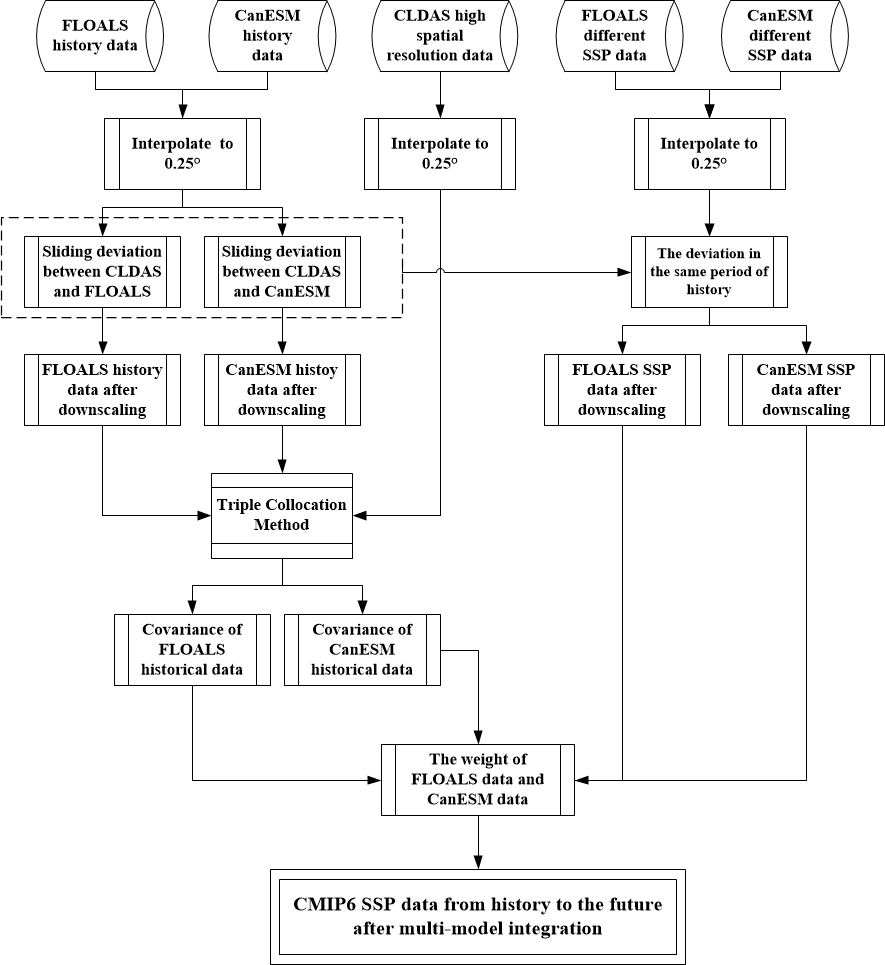


Fig. S2 The flowchart of spatial downscaling and model fusion for CMIP6 different models

Section 3. Effect of air temperature downscaling and model fusion

Taking temperature as an example, the original data of FGOALS and CanESMs models under different SSP scenarios, the results after downscaling of the sliding space, and the spatial comparison of the model fusion were drawn. As shown in the figure below, the original data of the two modes had obvious mosaic effects under different scenarios, especially in the western region, after sliding space downscaling and TC fusion, the mosaic effect was significantly reduced. And it can better reflect the trend that the temperature in China was gradually decreasing with the increase of elevation.


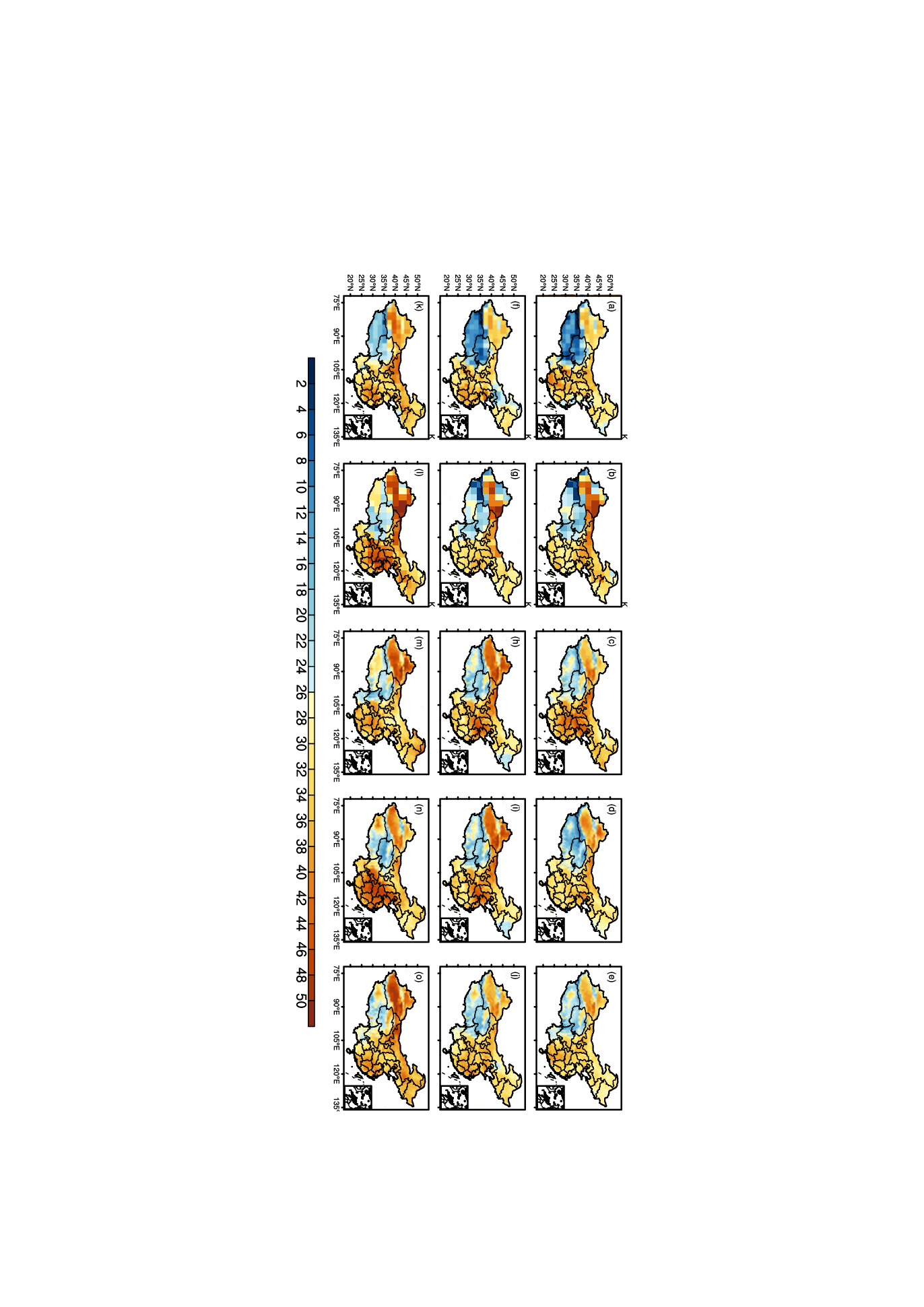


Fig. S3 The spatial comparison of original data, sliding spatial downscaling, and model fusion results under different SSP scenarios (taking the daily maximum temperature on August 1, 2100, as an example). The subplots from the top row to the bottom crow are daily maximum temperature under SSP126/SSP245/SSP585 respectively. The first and second columns are the original daily maximum temperature of FGOALS ( a, f, k) and CanESMs (b, g, l). The third and fourth columns are the sliding spatial downscaling daily maximum temperature of FGOALS ( c, h, m) and CanESMs ( d, i, n). The last column is the fusion result of daily maximum temperature (e, j, o). The figure was created using the NCAR Command Language 6.4.0 (https://www.ncl.ucar.edu)

Section 4. The threshold of HI and SI MHS in China


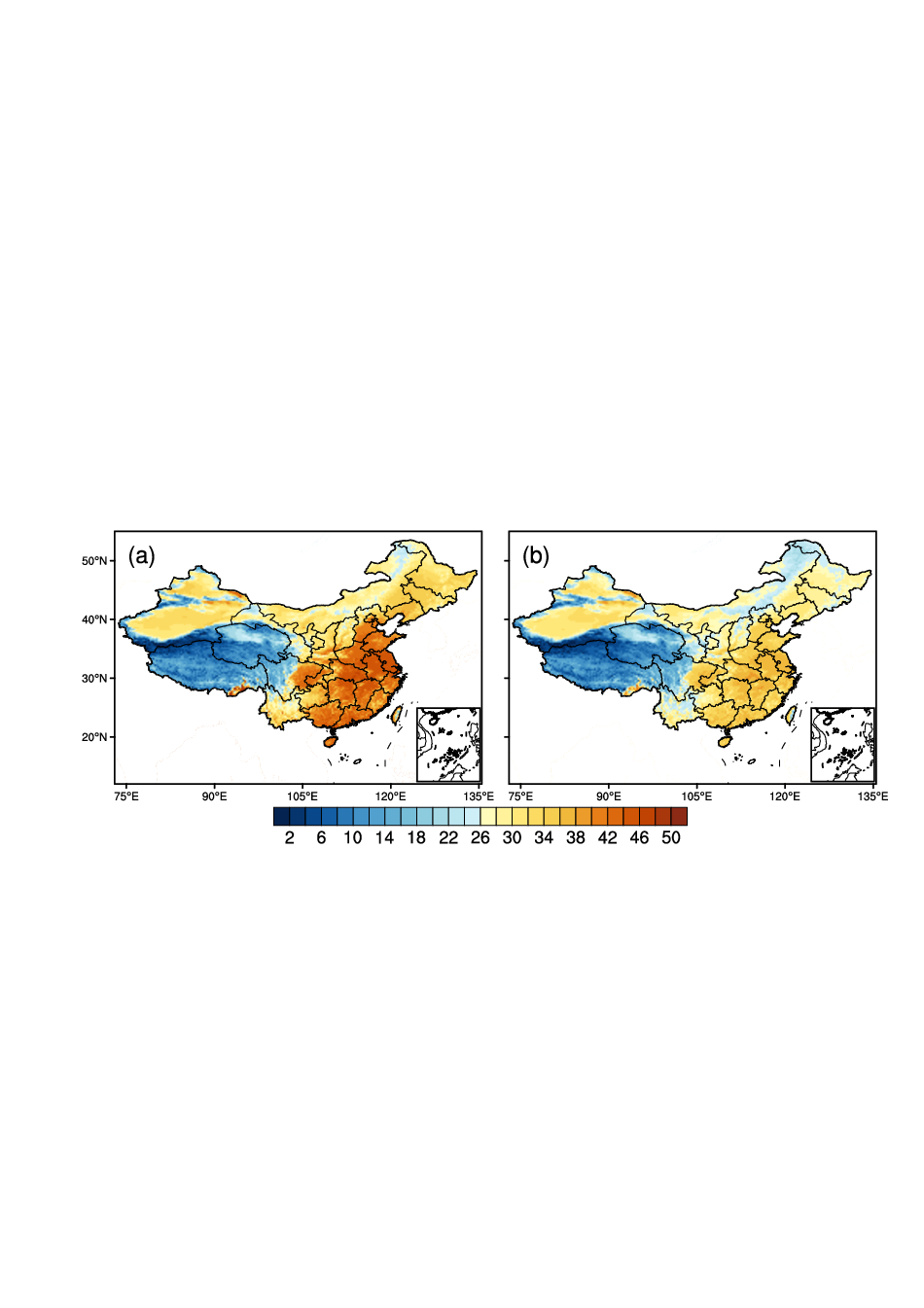


Fig. S4 The threshold of HI and SI MHS in China using the relative threshold and absolute threshold method. The subplot on the left is the threshold of HI (a), and the subplot on the right is the threshold of HI (b). The figure was created using the NCAR Command Language 6.4.0 (https://www.ncl.ucar.edu)

Section 5. The evaluation result of the relative humidity and wind speed of ERA5, GLDAS, CLDAS

The daily relative humidity of CLDAS ，GLDAS and ERA5 in the summer from 2008 to 2017 were interpolated to the in-situ sites by the bilinear interpolation method and compared with the observed relative humidity. The bias, RMSE, and Corr of ERA5, GLDAS, and GLDAS relative humidity with site observations were calculated, respectively, and the scatter plots were drawn. As shown in the figure below, the CLDAS relative humidity was closer to observations. The bias of CLDAS relative humidity was about 1%, while ERA5 was -16% and GLDAS was 3%. The RMSE of CLDAS relative humidity was about 4.3%, while ERA5 was 12% and GLDAS was 13%. The Corr of CLDAS was about 0.9, while the Corr of ERA5 was about 0.9 and GLDAS was between 0.63 and 0.8.


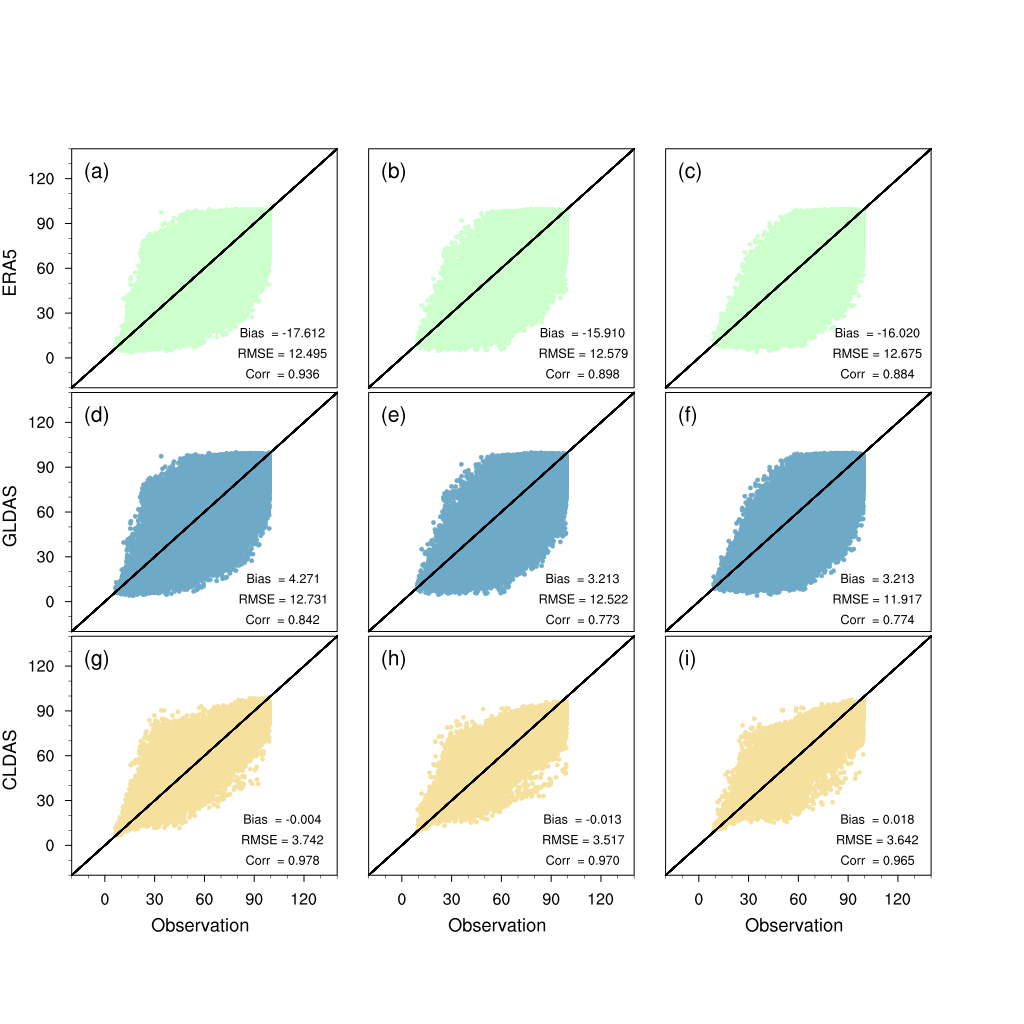


Fig. S5 The performance evaluation of ERA5, GLDAS, and CLDAS in describing the daily relative humidity changes during June, July, August from 2008 to 2017 based on bias, RMSE, and correlation analysis. The first column of subplots show evaluation results for June, the second and the third column are for July and August.

The daily wind speed of CLDAS, GLDAS and ERA5 in the summer from 2008 to 2017 were interpolated to the in-situ sites by the bilinear interpolation method and compared with the observed wind speed. The bias, RMSE, and Corr of ERA5, GLDAS, and GLDAS wind speed with site observations were calculated, respectively, and the scatter plots were drawn. As shown in the figure below, the CLDAS wind speed was closer to the observations. The bias of CLDAS wind speed was about -0.5m/s, while ERA5 was about 0.4m/s and GLDAS was about 0.6m/s. The RMSE of CLDAS wind speed was about 0.8m/s, while ERA5 was about 1m/s and GLDAS was about 1.15m/s. The Corr of CLDAS was about 0.77, while the Corr of ERA5 was about 0.65 and GLDAS was about 0.59.


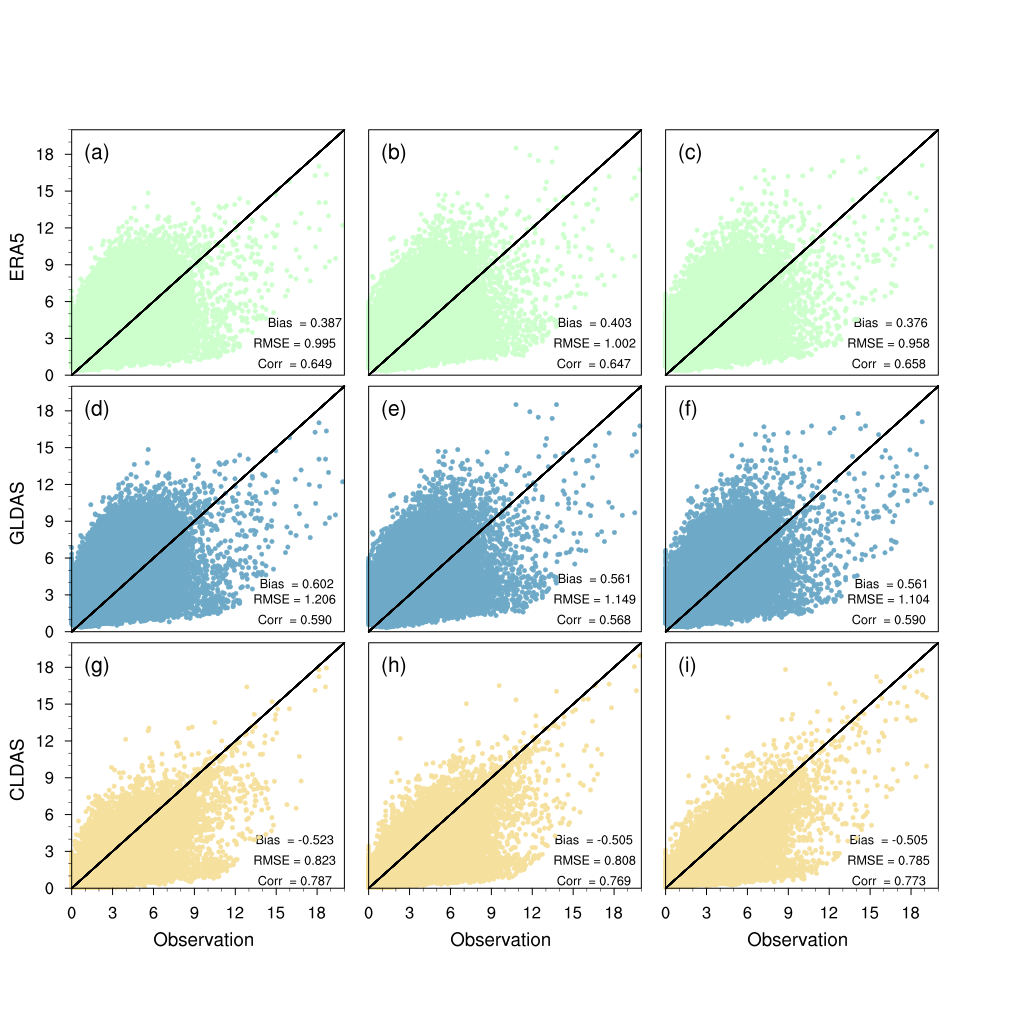


Fig. S6 The performance evaluation of ERA5, GLDAS, and CLDAS in describing the daily wind speed changes during June, July, August from 2008 to 2017 based on bias, RMSE, and correlation analysis. The first column of subplots show evaluation results for June, the second and the third column are for July and August.

Section 6. The relative humidity bias of FGOALS and CanESM

It can be seen from the bias of relative humidity that the original FGOALS data showed a large negative bias in northern and northwestern China and showed a large positive bias in southern China and the Qinghai-Tibet Plateau. CanESM presented a positive bias in most parts of China, while a negative bias in the northern part of Northeast China and the western part of the Qinghai-Tibet Plateau. The bias of FGOALS and CanESM data after SSD was significantly reduced, and the bias was -2 ~2 %. The bias of TC fusion results implied lower in some areas, such as in North China, Northeast China.


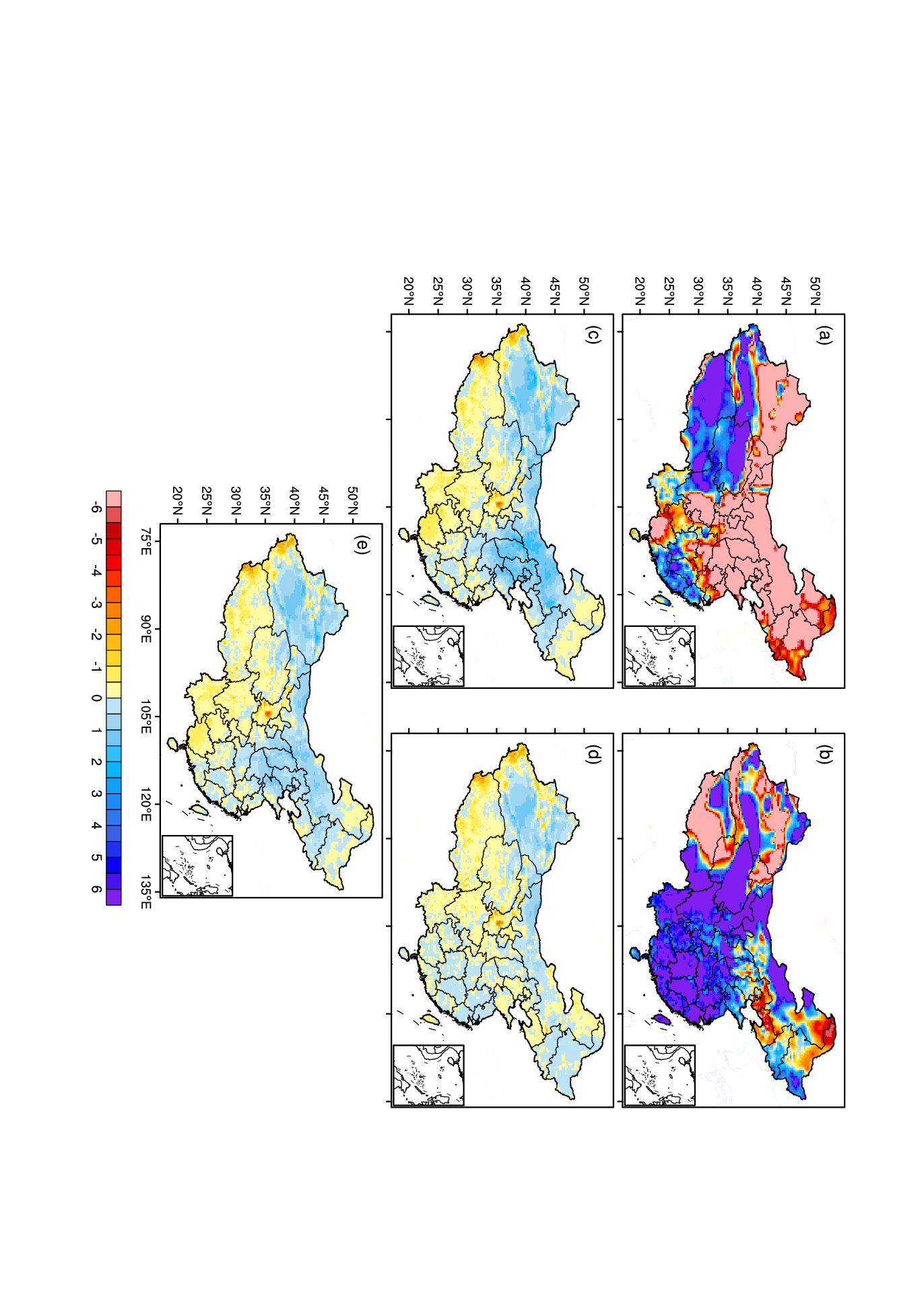


Fig. S7 The relative humidity bias of the original data, the SSD-based processed data, and model-based fusioned data (unit: %). (a) the original FGOALS outputs; (b) the original CanESM outputs; (c) SSD-based processed FGOALS outputs; (d) SSD-based processed CanESM outputs; (e) the model-fusion outputs. The figure was created using the NCAR Command Language 6.4.0 (https://www.ncl.ucar.edu)

Section 7. The temporal evolution of summer SI MHS frequency


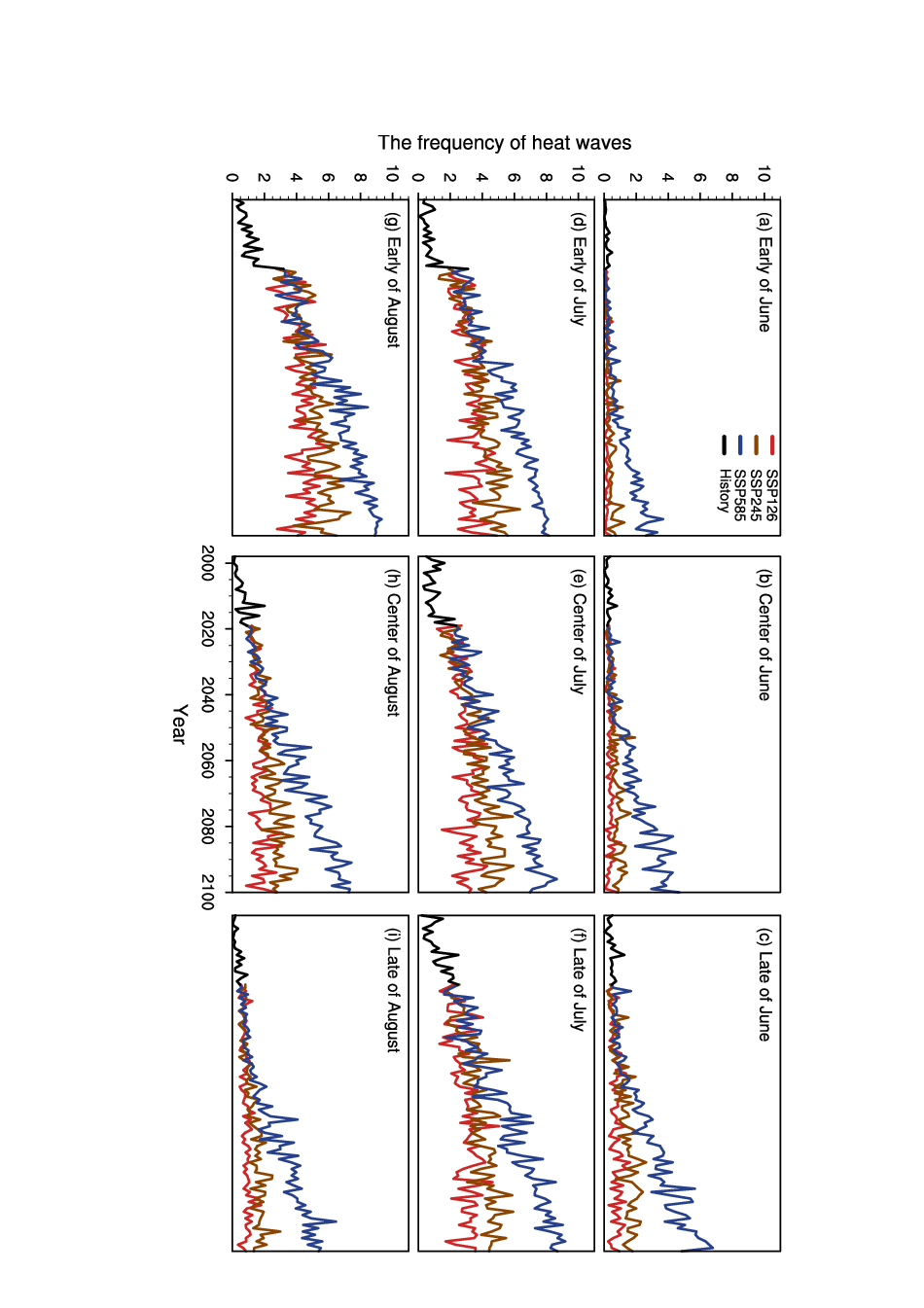


Fig. S8 Temporal evolution of summer SI MHS frequency under different scenarios in the early, middle, late of each month from 1998 to 2100. (a-c) the early, middle, late of June; (d-f) the early, middle, late of July, and (g-i) the early, middle, late of August. The solid black curves show a historical trend of the MHS-affected area. Red/brown/blue curves denote the future trend of the MHS-affected areas under SSP126, SSP245, SSP585 scenarios.

Section 8. The spatial evolution of the HI and SI MHS frequency

We analyzed the spatial evolution of the HI MHS frequency in 2000, 2010, 2020, 2040, 2060, 2080, and 2100 based on the CEEMDAN method. In a historical sense (Fig. s9a, S9b, and S9c), MHS in Northeast China and Qinghai were 10-15 MHS events more than that in 1998 and were 0-10 MHS events more than those in 1998 in other regions. In 2040, the MHS frequency in the Tibetan Plateau would increase by 10-55 MHS events, and in North China, Northeast China is less than 15, and in some places even reduced compared with 1998, while the MHS frequency in South China would increase by 0-15 MHS more than in 1998. As for MHS in southwest China, the MHS frequency under SSP126 and SSP245 scenarios would be similar to that in 2020, while MHS would gratly amplify under SSP585 when compared to that in 2020. In 2060, the MHS frequency under SSP126 would be subject to little change when compared to 2040. A higher frequency of MHS can be expected in Inner Mongolia, South China, and Southwest China under SP245, but less MHS would occur in the western part of the Qinghai-Tibet Plateau relative to 2040, while increased MHS frequency can be expected in the Qinghai-Tibet Plateau, Northeast China, North China, South China, and Southwest China when compared to 1998 and 2040 under SSP585. The frequency of MHS under SSP245 in Northeast China, western Inner Mongolia, and western Qinghai-Tibet Plateau would increase and would not change in the other places over China, while higher MHS frequency can be expected in 2060 under SSP585 in Northeast China and North China. In 2080, MHS frequency are similar to those in 2060 under SSP126 and SSP245, while MHS frequency would increase in most regions of China under the SSP585. In 2100, the MHS frequency in central Inner Mongolia and the Qinghai-Tibet Plateau would have a decreasing trend when compared to 2080 under the SSP126 scenario. For MHS frequency under the SSP245 scenario, MHS in Northeast China and the Qinghai-Tibet Plateau would have a slight lower frequency than in 2080 and little changes of MHS would be found in other areas of China. Except for South China and Southwest, the MHS frequency in other regions under SSP585 would be higher than in 2080, especially in the western regions of China. Simlar changing patterns can be found for SI MHS changes when compared to HI MHS changes (Fig.S10).


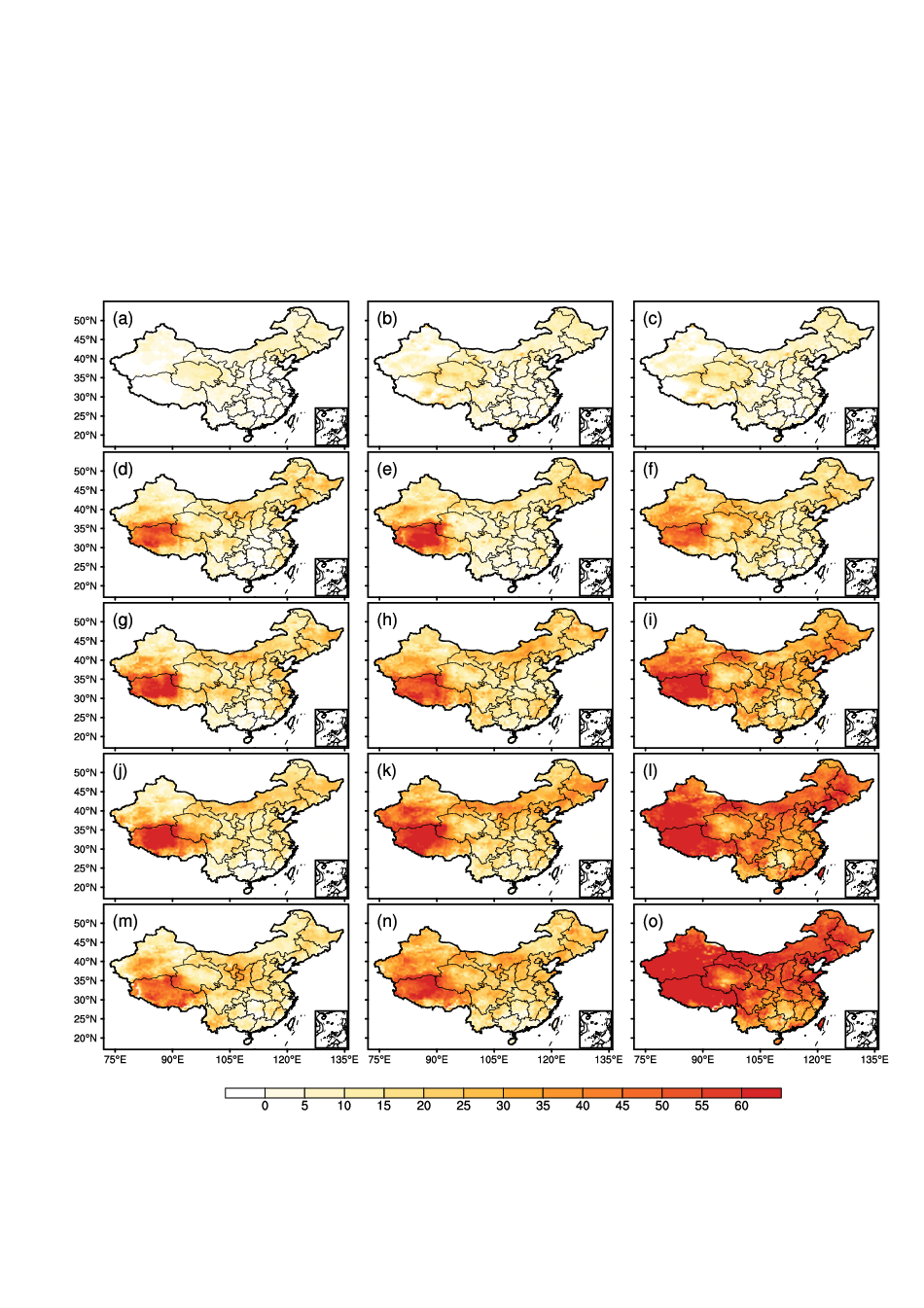


Fig. S9 Spatial evolution of HI MHS frequency based on the CEEMDAN method. (a), (b) and (c) show the HI MHS frequency in 2000, 2010, 2020, respectively, from a historical perspective. HI MHS frequency 2040 (d, e, f), 2060 (g, h, i), 2080 (j, k, l), 2100 (m, n, o). Column from the left to the right represents future HI MHS frequency under SSP126, SSP245, SSP585 scenarios. The figure was created using the NCAR Command Language 6.4.0 (https://www.ncl.ucar.edu)


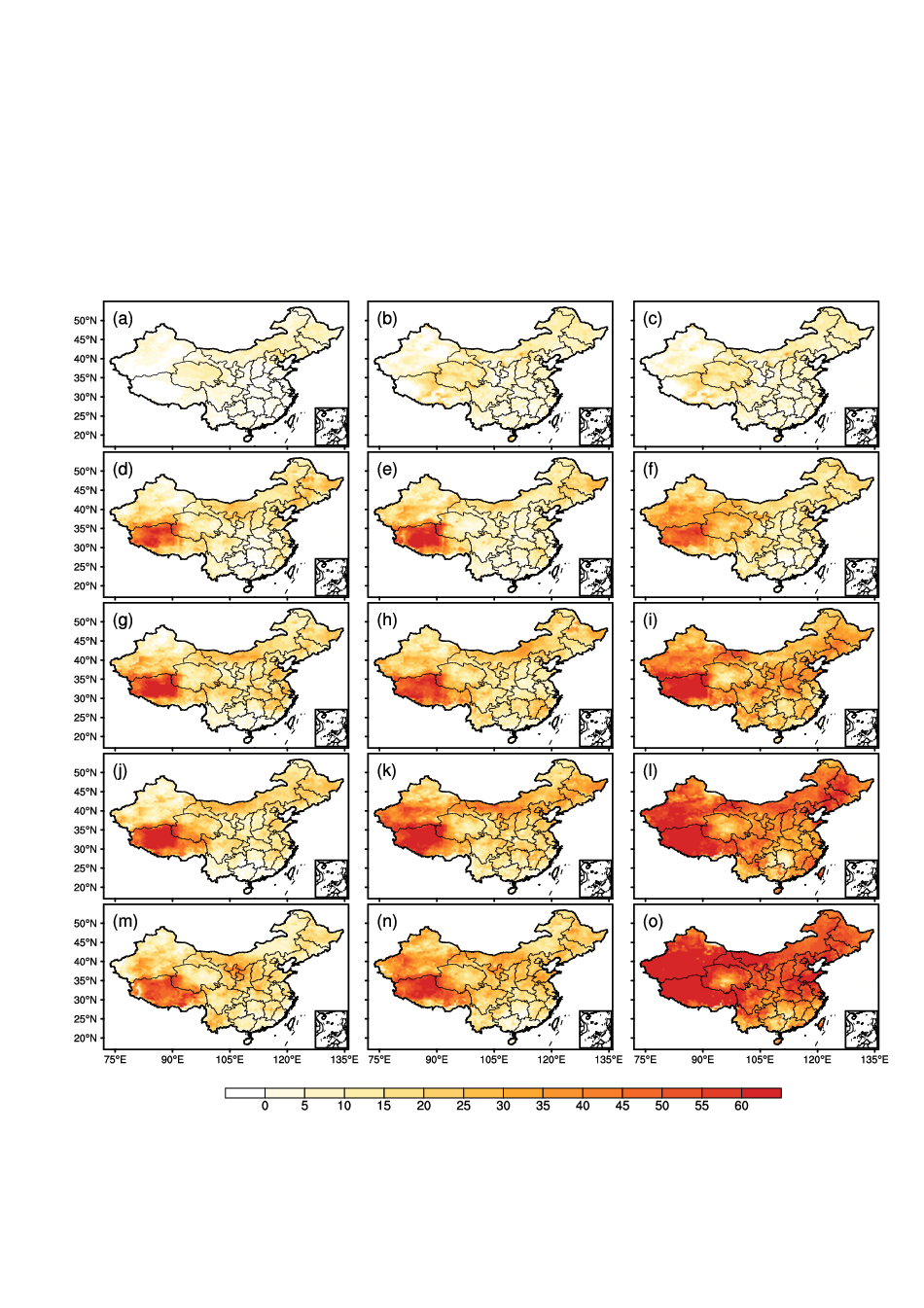


Fig. S10 The spatial evolution of the SI MHS frequency based on the CEEMDAN method. (a), (b) and (c) show the HI MHS frequency in 2000, 2010, 2020 respectively from the historical perspective. HI MHS frequency 2040 (d, e, f), 2060 (g, h, i), 2080 (j, k, l), 2100 (m, n, o). From the left to the right column represents future HI MHS frequency under SSP126, SSP245, SSP585 scenarios. The figure was created using the NCAR Command Language 6.4.0 (https://www.ncl.ucar.edu)

Section 9. The spatial evolution of the SI MHS intensity


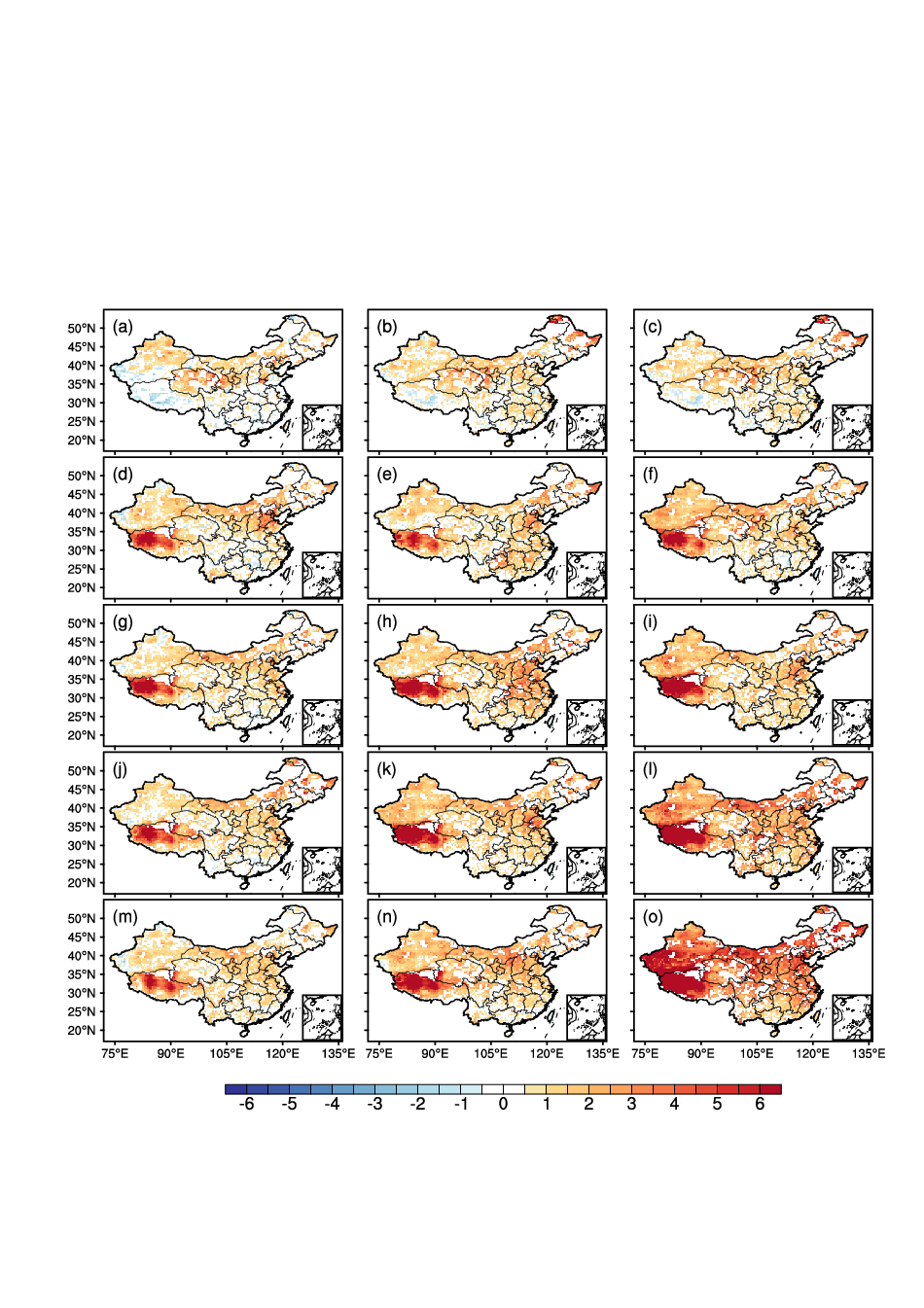


Fig. S11 The spatial evolution of the SI MHS frequency based on the CEEMDAN method. (a), (b) and (c) show the HI MHS frequency in 2000, 2010, 2020 respectively from the historical perspective. HI MHS frequency 2040 (d, e, f), 2060 (g, h, i), 2080 (j, k, l), 2100 (m, n, o). From the left to the right column represents future HI MHS frequency under SSP126, SSP245, SSP585 scenarios. The figure was created using the NCAR Command Language 6.4.0 (https://www.ncl.ucar.edu)

Section 10. The spatial evolution of the HI and SI MHS duration

The spatial evolution of the HI heatwave duration in 2000, 2010, 2020, 2040, 2060, 2080, and 2100 based on the CEEMDAN method (Fig. S12) showed that HI MHS duration of northeast and west China during 2000, 2010, and 2020 was 2-6 days longer than that during 1998. Evident lengthening of the HI MHS duration was found in the northern parts of Northeast China and Qinghai Province. Lengthening HI MHS duration was detected in 2040, when compared to that in 1998, in North China, Northeast China, West China, and Qinghai-Tibet Plateau under SSP126, SSP245, SSP585 scenarios would increase compared with 1998, and remarkable lengthening HI MHS duration was observed in the western part of the Qinghai-Tibet Plateau, being followed by HI MHS duration in the northeast and western China. Comparison of HI MHS duration between 1998 and 2060 under SSP585 indicated profound lengthening HI MHS duration in Northeast China, North China, Northwest China, and the Qinghai-Tibet Plateau. In general, lengthening of the HI MHS duration was coming to be larger from SSP126, SSP245 to SSP585 and the evident lengthening of the HI MHS duration was found mainly in the Northeast China, North China, Northwest China, and the Qinghai-Tibet Plateau. These regions were arid and semi-arid in climate types with fragile ecosystem and water shortage. Lenghthening MHS duration in arid and semiarid regions should arouse considerable human concerns.


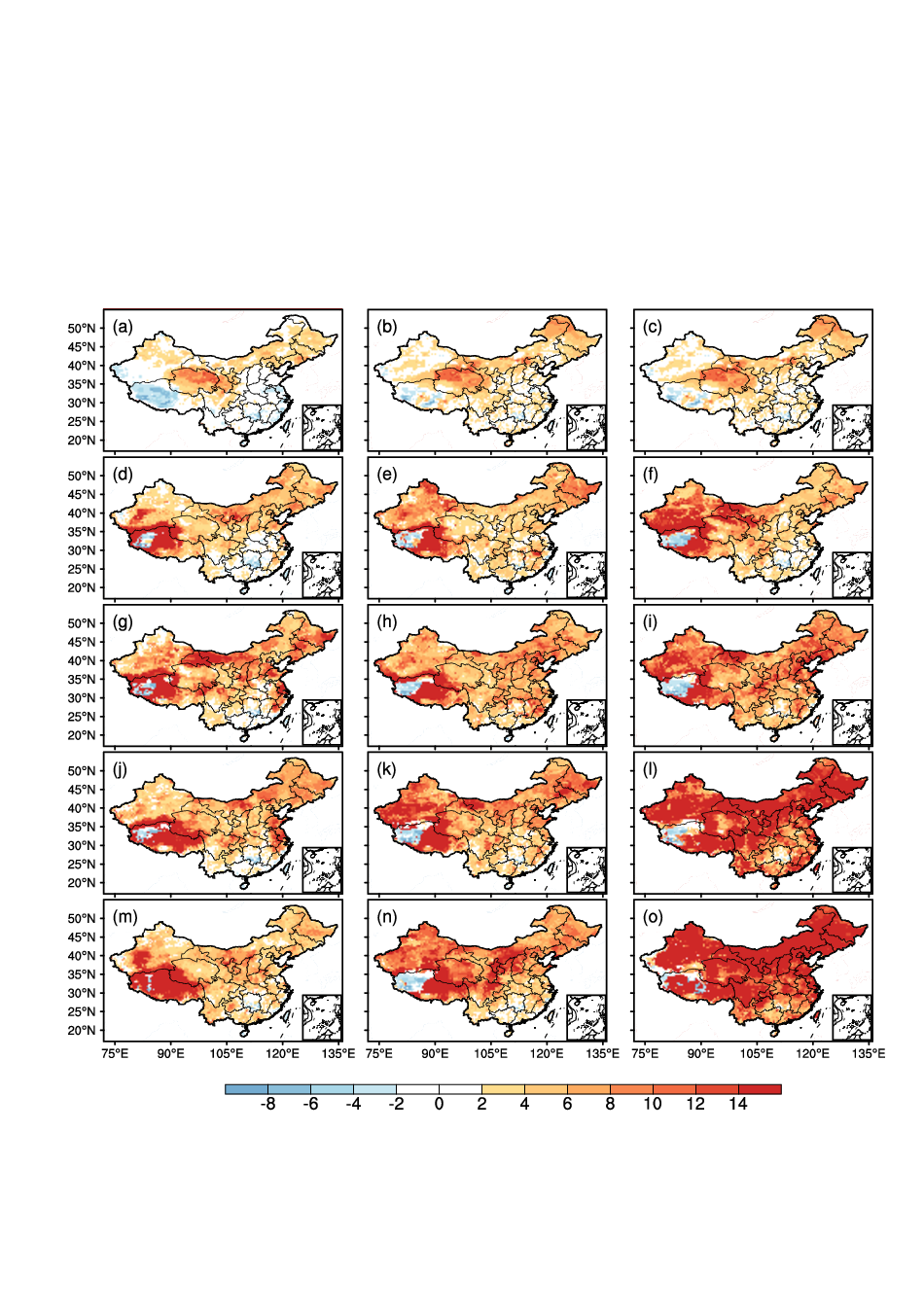


Fig. S12**.** Spatial evolution of the HI MHS duration based on the CEEMDAN method. (a), (b) and (c) show HI MHS duration in 2000, 2010, 2020. Spatial pattern of HI MHS duration during 2040 (d, e, f), 2060 (g, h, i), 2080 (j, k, l), 2100 (m, n, o) respectively and column from left column to right shows HI MHS duration under SSP126, SSP245, SSP585 scenarios. The figure was created using the NCAR Command Language 6.4.0 (https://www.ncl.ucar.edu)


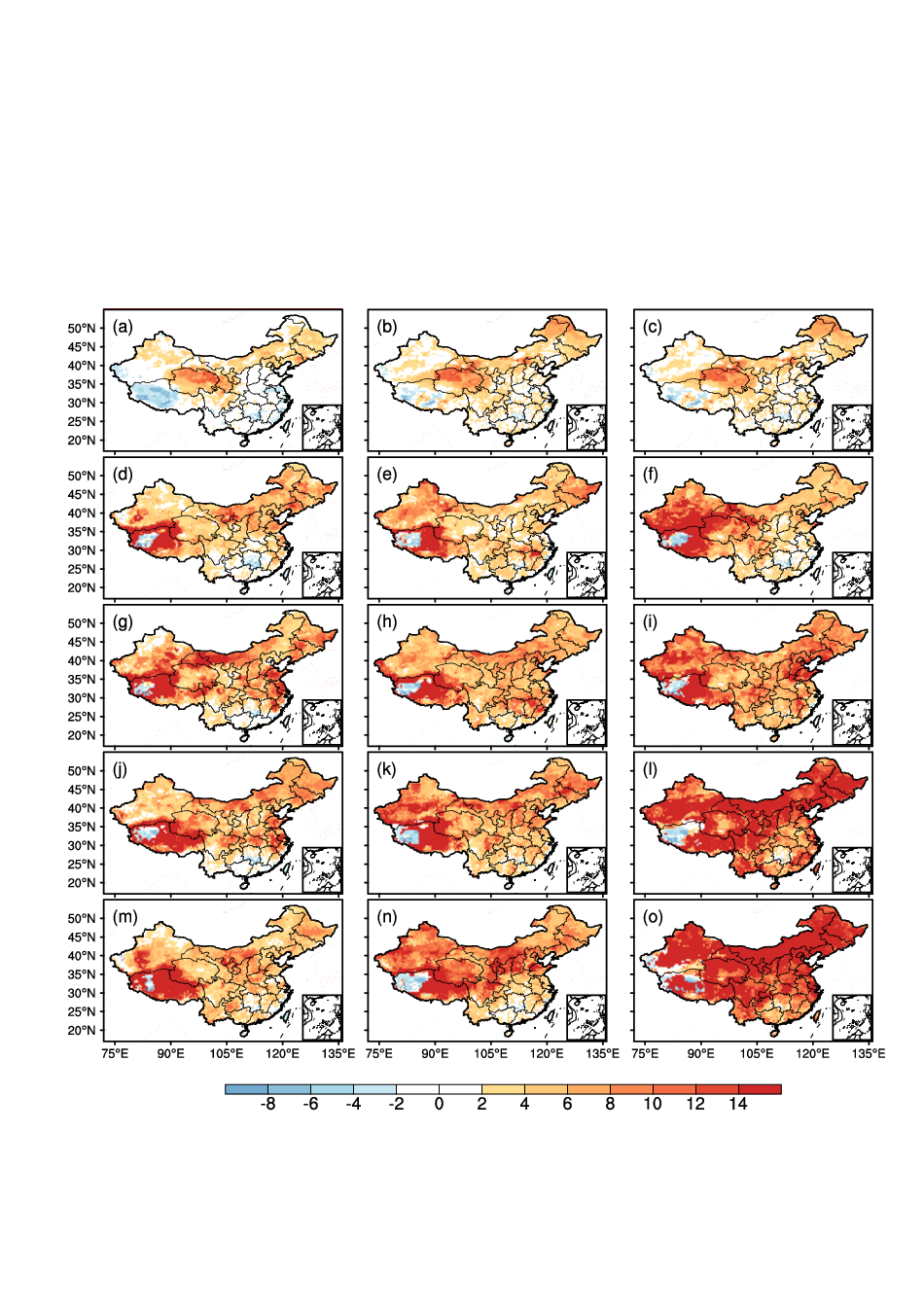


Fig. S13 The spatial evolution of the SI MHS duration based on the CEEMDAN method. (a), (b) and (c) show HI MHS duration in 2000, 2010, 2020. Spatial pattern of HI MHS duration during 2040 (d, e, f), 2060 (g, h, i), 2080 (j, k, l), 2100 (m, n, o) respectively and from left column to right column show HI MHS duration under SSP126, SSP245, SSP585 scenarios. The figure was created using the NCAR Command Language 6.4.0 (https://www.ncl.ucar.edu)

Section 11. The temporal evolution of the HI and SI MHS-afffected areas

Here we analyzed changes of summer HI and SI MHS-afffected areas from 1998 to 2100 (Fig. S14). During 1998 to 2020, the MHS-affected regions gradually increased with an average annual increase of 0.399% for HI and 0.479% for SI. Under the scenarios considered in this study, the HI and SI-based MHS-affected area under SSP126 would increase and then would be in moderate changes around 2060. Specifically, the HI-based MHS-affected areas would increase by 0.08%/year from 2021 to 2060, and the SI-based MHS-affected areas would increase by 0.07%/year from 2021-2060. However, the HI MHS-affected areas under the SSP245 showed a larger increase, being 0.249%/year and 0.24%/year SSP245 for HI and SI respectively. The HI and SI MHS-affected areas under SSP585 were higher than that under SSP126 and SSP245, with an average increase of 0.669%/year and 0.658%/year.


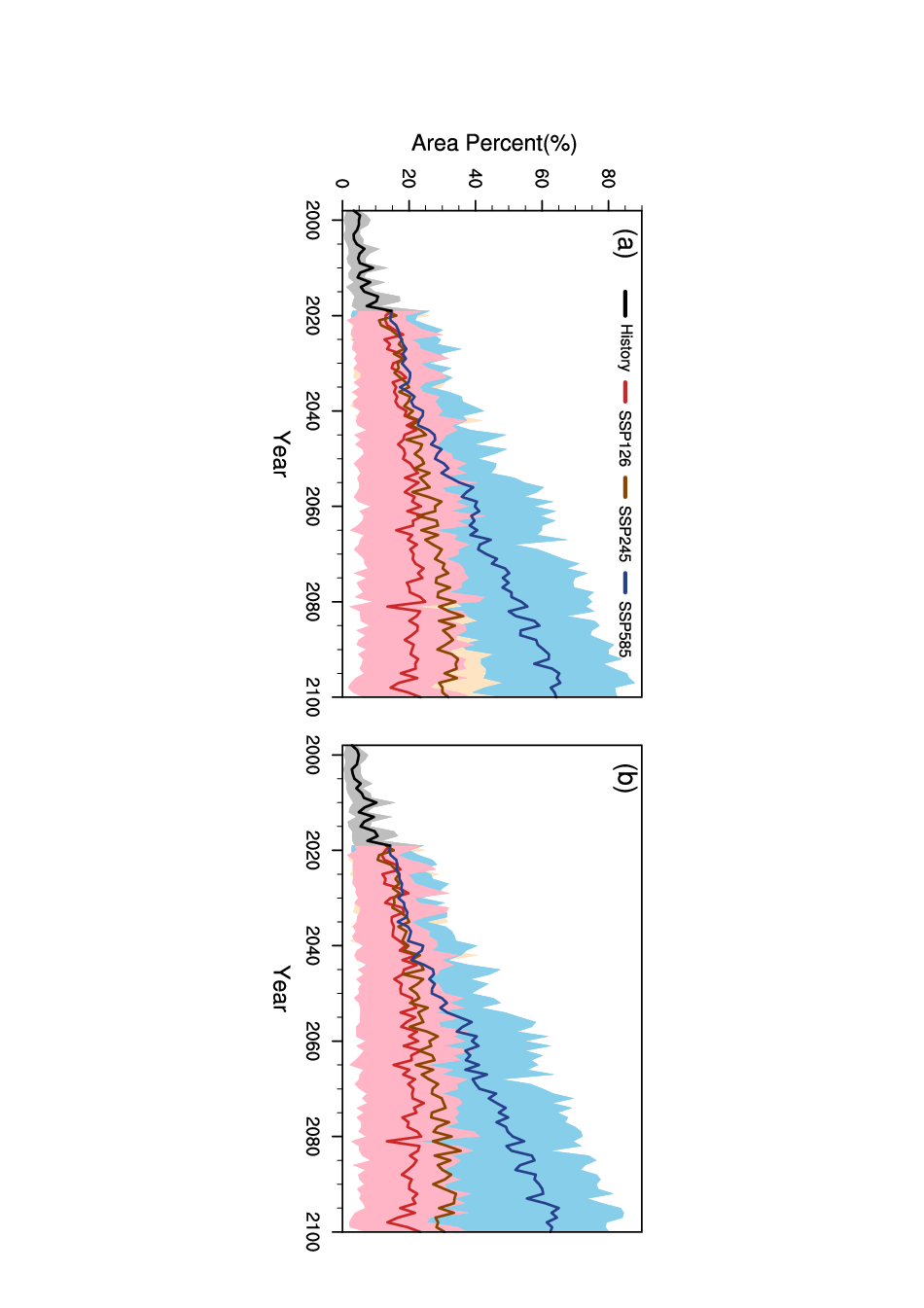


Fig. S14 Temporal tendency of the summer MHS-affected areas during the period of 1998 to 2100. (a) HI-based; (b) SI-based MHS. The solid black curves show the tendency of the historical summer MHS-affected areas. Red/brown/blue curves denote the tendency of the future summer MHS-affected areas under SSP126/SSP245/SSP585 scenarios. Shaded areas show the pattern distribution between the 25th and 75th quartiles.
